# A Microfiber‐Reinforced Janus Hydrogel E‐Skin With Recyclable Feature for Multimodal Sensing and Gender‐Specific Physiological Monitoring

**DOI:** 10.1002/advs.202520336

**Published:** 2025-12-22

**Authors:** Yarong Ding, Yufeng Li, Shaozhe Tan, Jiachun Sun, Xu Yang, Xuesi Zhang, Zhenhua Lin, Zhenyu Li, Yue Hao, Yannan Liu, Yingchun Li, Jingjing Chang

**Affiliations:** ^1^ State Key Laboratory of Wide‐Bandgap Semiconductor Devices and Integrated Technology, Faculty of Integrated Circuit Xidian University Xi'an P. R. China; ^2^ Shaanxi Key Laboratory of Degradable Biomedical Materials, School of Chemical Engineering Northwest University Xi'an P. R. China; ^3^ Advanced Interdisciplinary Research Center for Flexible Electronics, Academy of Advanced Interdisciplinary Research Xidian University Xi'an P. R. China

**Keywords:** dual‐mode, ionotronic conductivity, janus hydrogel, physiological monitoring, tough hydrogel

## Abstract

Hydrogel‐based wearable electronics hold great promise for physiological monitoring, yet their application in privacy‐sensitive regions remains constrained by the simultaneous demands of ultrathin form factors, mechanical robustness, multimodal sensing, and long‐term stability. Inspired by dragonfly wings, this study develops a gelatin hydrogel e‐skin reinforced with polyurethane (PU) microfibers, featuring ultra‐thinness (7.15 µm), high strength (55.62 MJ m^−3^), and high sensitivity (GF = 2.52, TCR = 3.5%°C^−1^). Its controlled binary heterogeneous structure ensures asymmetric adhesion and long‐term skin conformability. A deep eutectic solvents (DES)‐induced ion‐electron dual‐conducting system enhances conductivity by 13 times while improving flexibility, thereby boosting sensing performance. This sensor boasts biocompatibility, antibacterial properties, transparency, freeze resistance and recyclability. It enables high‐precision continuous monitoring and achieves multimodal signal decoupling via finite element design. Integrated with flexible circuits and wireless modules, it supports non‐invasive at‐home tracking of privacy signals during pregnancy and erectile function. This work offers an intelligent solution for high‐precision monitoring in precision and personalized healthcare.

## Graphical abstract

Hydrogel‐based wearable electronics hold great promise for physiological monitoring in privacy‐sensitive regions. In this study, a polyurethane (PU) microfiber‐reinforced gelatin hydrogel e‐skin is developed, boasting multiple advantages such as ultra‐thinness, high toughness, and long‐term skin conformability. After being decoupled, it can be effectively applied for non‐invasive at home privacy signal monitoring.

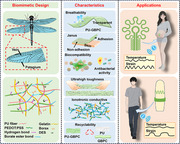

## 1 Introduction

Home‐based reproductive health monitoring is of paramount significance for the early detection of health risks and the delivery of precision health management [1, 2]. For pregnant women, physiological monitoring can assess fetal development and preterm birth risk through abdominal temperature and abdominal circumference strain (e.g., uterine contractions, fetal movements) [1]. Male erectile dysfunction (ED) is a potential marker for cardiovascular diseases and diabetes [3, 4]. Currently, monitoring for these two privacy‐sensitive conditions is primarily reliant on invasive devices or subjective questionnaires [1, 5]. Existing physiological monitoring devices still suffer from issues including limited indicators, poor stability, wear discomfort, and complex operation, hindering the implementation of home‐based self‐monitoring [6, 7]. Thus, there is an urgent unmet need for multi‐modal, highly biocompatible dynamic monitoring platforms tailored to home‐use scenarios.

Hydrogels are widely recognized as ideal flexible sensing materials [8]; however, most current hydrogel‐based sensors are either limited to single‐modal detection or plagued by severe signal crosstalk in multimodal sensing scenarios [9]. To address cross‐sensitivity, decoupled sensing mechanisms, including piezoresistive, capacitive, triboelectric, pyroelectric, and thermoresistive approaches, have been developed [10, 11, 12, 13]. Nevertheless, most of these strategies rely on complex algorithms, which result in high power consumption and compromised real‐time sensing capabilities, thereby limiting their practical applicability [9]. Given that privacy‐sensitive body regions demand precise conformity to complex curved surfaces, the development of intrinsically decoupled multi‐modal hydrogel sensors remains a critical technical bottleneck.

Beyond multimodal sensing capabilities, the mechanical robustness of hydrogels is also critical for withstanding repeated stretching induced by abdominal distension during pregnancy and penile tumescence in males [14, 15]. Fiber‐reinforced double‐network (DN) designs have been shown to significantly enhance the stretchability of composite hydrogels, while tailoring fiber diameter enables a balance between high mechanical strength and ultra‐thinness [16]. To enhance hydrogel conformity with privacy‐sensitive body sites, catechol‐containing compounds (e.g., polydopamine, tannic acid) are commonly incorporated into hydrogel matrices [17, 18]. However, conventional double‐sided adhesive configurations often exhibit excessive adhesion to the external surfaces of privacy‐sensitive regions, which inadvertently interferes with the accurate acquisition of physiological signals [14, 16, 19, 20, 21, 22, 23, 24]. In recent years, hydrogels with asymmetric adhesiveness have emerged as promising candidates for applications in biomedicine and flexible electronics [25, 26, 27, 28]. Nevertheless, Janus hydrogels, characterized by asymmetric designs, often suffer from critical limitations, including weak interfacial bonding or bandgap mismatch. This results in the formation of high‐resistance interfaces, further restricting their deployment on body surfaces.

To achieve high signal‐to‐noise ratio multi‐mode sensing, hydrogel matrices are often filled with conductive fillers [25] and intrinsically conductive polymers, such as poly(ethylene dioxythiophene): poly(styrene sulfonate) (PEDOT:PSS) [26]. However, PEDOT:PSS exhibits low conductivity and high rigidity [27] owing to inherent structural defects, a limitation that necessitates secondary doping. Ionic liquids (ILs) can enhance the electrical conductivity and stretchability of PEDOT:PSS [29], however, many ILs suffer from toxicity and poor degradability, necessitating replacement with natural biomaterials [30]. Deep eutectic solvents (DES), as environmentally friendly alternatives to ILs, exhibit high viscosity, non‐volatility, non‐toxicity and degradability [31]. Although significant progress has been made in the conductivity and environmental stability of DES‐doped PEDOT:PSS [32], its utility in monitoring complex reproductive physiological signals remains largely unexplored.

**SCHEME 1:**
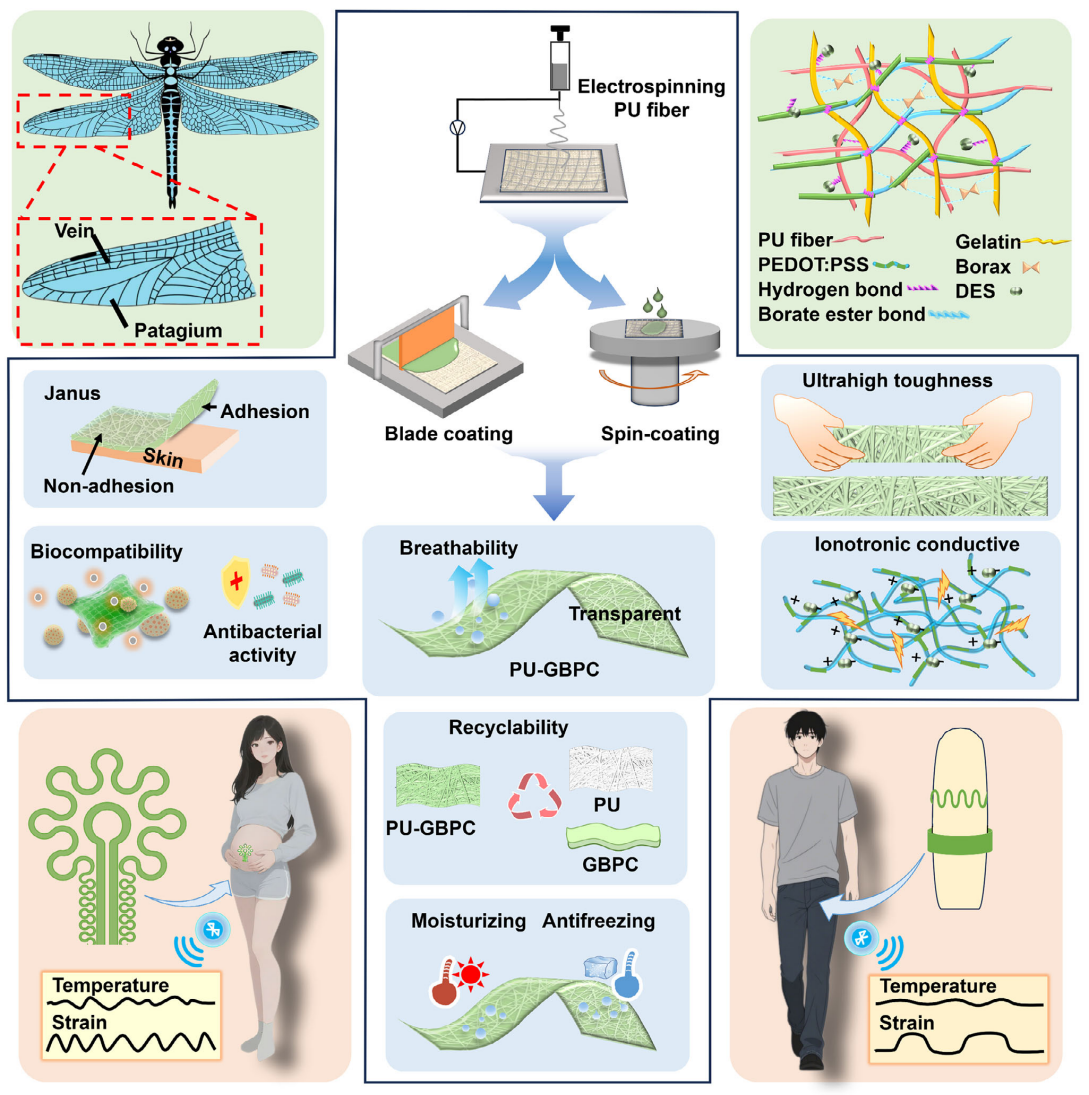
Schematic diagram of the inspiration, preparation process, mechanism, performance, and application of the PU‐GBPC hydrogel sensor.

Inspired by the microarchitecture of dragonfly wings, we designed a dual‐mode hydrogel sensor (PU‐GBPC) (Scheme 1). Its core advantage lies in the combination of an ultra‐thin structure and high toughness reinforced by polyurethane (PU) fibers. Meanwhile, the synergistic interpenetration of PU microfibers and hydrogel networks, mediated by abundant hydrogen bonding and hydrophobic interactions, endows the sensor with an integrated suite of properties including breathability, antibacterial activity, biocompatibility, recyclability, and resistance to drying. The incorporation of DES‐doped PEDOT:PSS establishes an ion‐electron dual‐conductive network, achieving high strain/temperature sensitivity over a wide range as well as cyclic stability. Importantly, the partial penetration of PU fibers forms an asymmetrically adhesive Janus structure, which enables long‐term monitoring of signals from body surfaces and mechanical joints. To eliminate signal interference, PU‐GBPC is cut into a double‐loop serpentine structure and connected to a flexible circuit, thereby constructing an intelligent monitoring system for private areas. Abnormal uterine activity in the abdomen of pregnant women and erectile status of the male penis can be monitored through dual‐modal strain and temperature signals. After clinical validation, this holds promise for enabling disease type identification in different scenarios and realizing home‐based privacy‐sensitive monitoring.

## 2 Results and Discussion

### 2.1 Preparation and Characterization of PU‐GBPC Hydrogel Sensors

**FIGURE 1:**
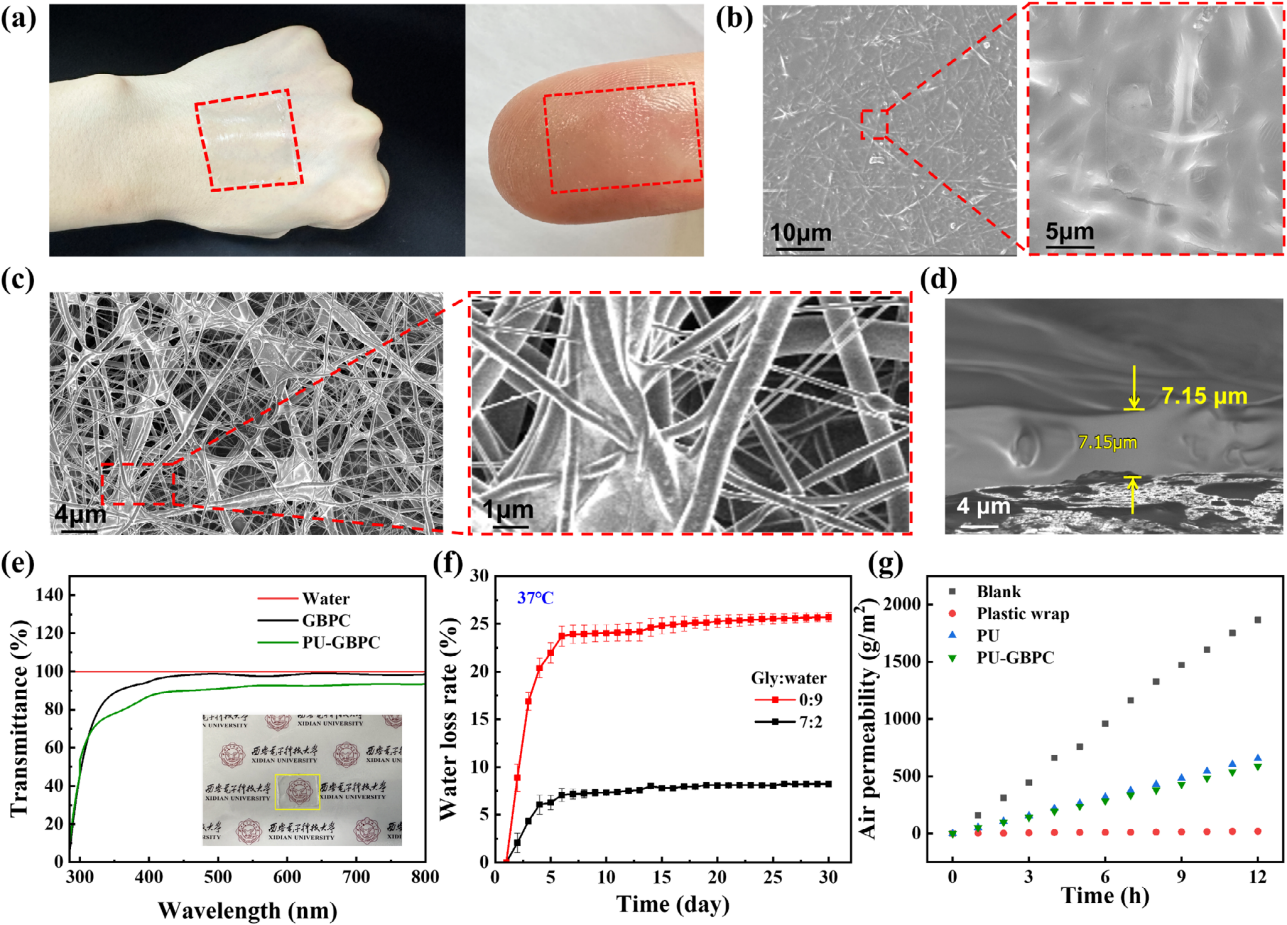
Characterization of the PU‐GBPC hydrogel sensor. a) Excellent skin conformability of the PU‐GBPC hydrogel. b) SEM images of the front gel surface and its local magnification. c) SEM images of the back fiber surface and its local magnification. d) Cross‐sectional SEM image. e) Light transmittance in the wavelength range of 300 nm to 800 nm, with the inset being an optical photograph of the transmittance. f) Water loss rate in a constant temperature (37°C) environment for 30 days. g) Air permeability of the PU‐GBPC hydrogel sensor and the control group for 12 consecutive hours.

In the composite hydrogel sensor, gelatin serves as the hydrogel network matrix. The excellent multifunctionality endowed by its unique amino acid sequence endows it with significant advantages in dynamic adaptability, interfacial adhesion, and mechanical toughness. Gelatin‐based hydrogel network relies on abundant hydrogen bonds and hydrophobic interactions, which synergistically penetrate to form a stable and distinctive structure [33]. By spin‐coating or blade‐coating the hydrogel precursor solution onto PU fibers, the composite hydrogel film (PU‐GBPC) can be obtained (Figure S1), whereas, GBPC refers to the pure hydrogel film without PU fibers. This biomimetic strategy of “rigid framework‐flexible matrix integration” inspired by dragonfly wings enables the GBPC hydrogel to achieve moderate interpenetration at the nodes and gaps of the PU fiber network, thereby further enhancing the performance of the composite hydrogel. The PU‐GBPC membrane exhibits excellent conformability when applied to the skin, and its thinness, light weight, and transparency allow it to embed within fingerprint textures, making it a promising candidate for e‐skin applications (Figure 1a). SEM imaging reveals that the front side of the composite hydrogel is hydrogel‐dominated with fully embedded PU fibers, enabling the polymer network and nanofibers to deform synergistically under tensile stress and prevent delamination (Figure 1b). In contrast, the back side is fiber‐dominated with partial hydrogel penetration, which enhances tensile strength (Figure 1c; Figure S2). Cross‐sectional SEM images indicate a composite thickness of only 7.15 µm (Figure 1d).

Additionally, the effects of PEDOT:PSS and Choline chloride (ChCl) on enhancing the hydrogel's conductivity were investigated. The results in Figure S3a, b demonstrate that the conductivities of electron‐only conduction and ion‐only conduction are 0.11 S/m and 0.42 S/m, respectively, which are 2.2 times and 8.4 times higher than those of the pristine hydrogel. This indicates that the addition of PEDOT:PSS has a negligible effect on the electrical conductivity of the hydrogel, while that of ChCl significantly enhances it. This is because the formed DES widens charge transport channels, thereby significantly enhancing ionic mobility. When the content of DES (glycerol + ChCl) reaches a certain level, the conductivity tends to stabilize. Excessive DES weakens physical crosslinking points such as hydrogen bonds, leading to the loosening of the hydrogel network structure, decreased modulus, and reduced mechanical strength. More importantly, the increased glycerol content makes it prone to evaporation, resulting in gradual embrittlement of the material. So co‐doping of PEDOT:PSS and ChCl yields the optimal GBPC2, with a conductivity as high as 0.66 S m^−1^, which is 13 times higher than that of the original hydrogel. (Table S1).

To validate the strain‐responsive conductivity of PU‐GBPC, a 1 cm × 3 cm sample was integrated into a flower‐patterned light‐emitting diode (LED) circuit. Upon stretching the hydrogel, the LED brightness gradually diminished (Figure S4 and Video S1), confirming a strong correlation between hydrogel deformation and sensor conductivity. Fourier transform infrared spectroscopy (FTIR) was used to analyze the interactions and structural formation of the GBPC hydrogel (Figure S5). The peak shift from choline chloride to the PU‐GBPC hydrogel confirms the formation of DES [34]. The peak at 1230 cm^−1^ originates from the asymmetric stretching vibration of B‐O‐C, which proves the formation of borate ester bonds [35]. The blue shift of the characteristic broad absorption band at 3092–3570 cm^−1^, corresponding to the symmetric stretching of O‐H, indicates the existence of strong hydrogen bonds between components [36]. When applied to skin, the hydrogel is as transparent as biological tape (Figure 1e inset). The transmittance of GBPC reaches 98.9% in the visible light range of 500–700 nm, while that of the PU‐GBPC hydrogel membrane is slightly lower. Beyond transparency, anti‐desiccation performance is also critical for long‐term health monitoring. PU‐GBPC contains abundant glycerol, which effectively locks water [37], resulting in a water loss rate of <8.5% after 30 days at 37°C, whereas glycerol‐free PU‐GBPC loses 23.72% of its water within 6 days (Figure 1f). Air permeability of the PU‐GBPC hydrogel film was further evaluated (Figure 1g), with a measured water vapor transmission rate (WVTR) of 50.66 g m^−2^ day^−1^. Embedding the hydrogel within PU fibers reduces pore size, leading to slightly lower air permeability compared with pure PU fiber membranes (Figure S6).

### 2.2 Mechanical Properties of PU‐GBPC Hydrogel Sensors

**FIGURE 2:**
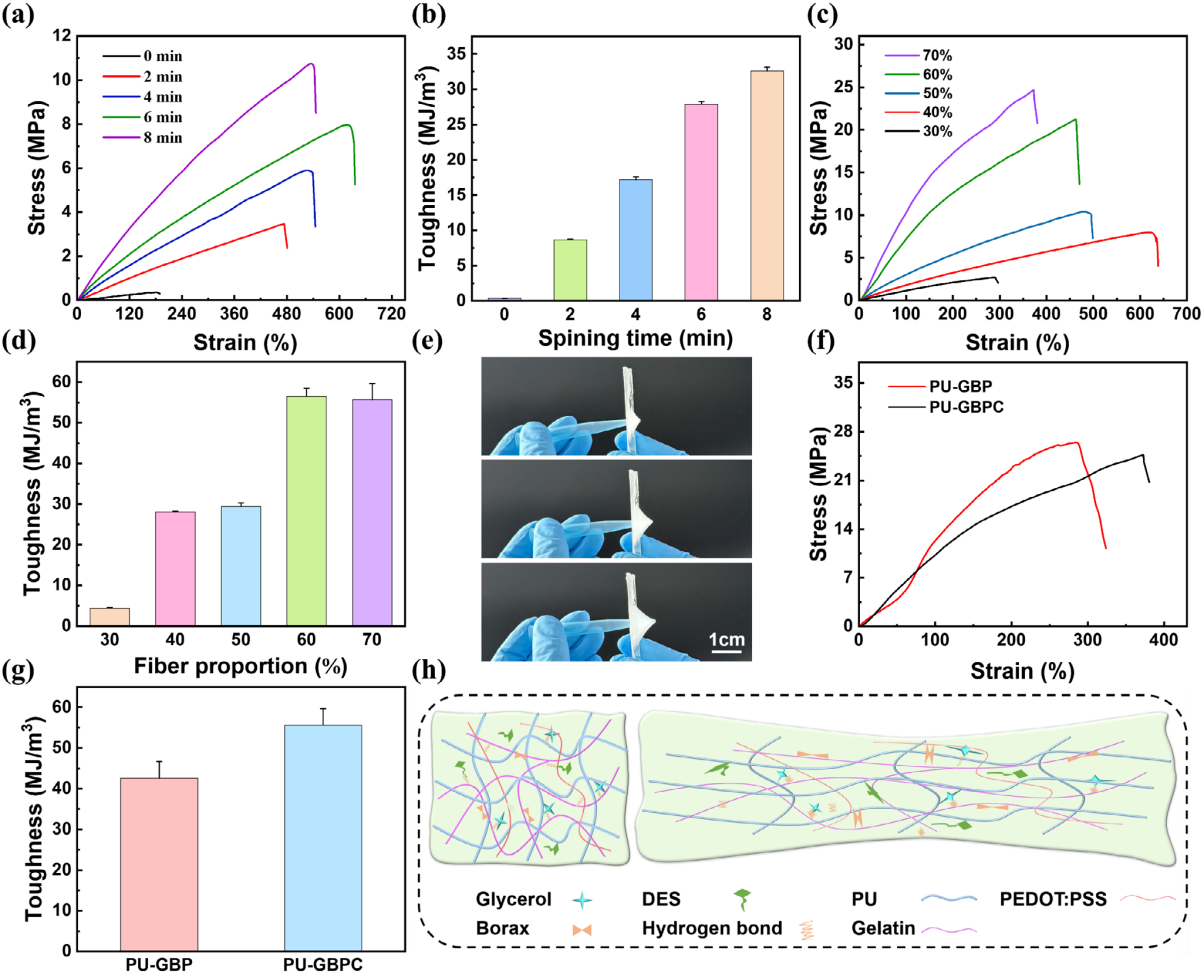
Mechanical properties of the PU‐GBPC hydrogel sensor. a) Stress–strain curves under different spinning times. b) Comparison of toughness under different spinning times. c) Stress–strain curves at different fiber weight fractions. d) Toughness comparison at different fiber weight fractions. e) The PU‐GBPC composite hydrogel exhibits excellent puncture resistance. f) Stress–strain curves of different components. g) Comparison of toughness of hydrogels with different components. h) Schematic diagram of internal network stretching.

Compared with pristine hydrogel membranes, the PU fiber‐reinforced composite architecture achieves a twofold enhancement in mechanical performance, a critical improvement for sustaining repeated deformation during physiological monitoring [38]. The thickness of PU fibers governs their interaction with the hydrogel matrix and thereby dictates the overall mechanical behavior of the composite [14, 39]. To prepare an ultra‐thin composite hydrogel, we adopted the constant‐speed spin‐coating method during the optimization process. By regulating the spin‐coating speed to match the acceleration and precisely controlling the rheological consistency of the precursor solution, the resulting samples exhibit excellent uniformity. First, keep the gel content constant while varying the thickness of the fibers on the substrate. As shown in Figure 2a and Figure S7, the incorporation of PU fibers markedly increased both tensile strength and toughness relative to pure GBPC. With spinning time extended from 2 to 8 min, the elastic modulus and toughness gradually increased, consistent with the stronger reinforcing effect of thicker fibers (Figure 2b; Figure S8). However, when the electrospinning time exceeds 6 min, the gel layer and the fiber layer fail to form an interpenetrating interface with a continuous stress transition gradient; instead, a distinct physical interface with weak bonding is formed. Under external tensile loading, stress cannot be effectively transferred or dispersed between the two layers, resulting in a decrease in elongation at break (Figure S9). At low deposition times (2‐4 min), sparse fiber deposition produced an open network, leaving the composite hydrogel‐dominated. At 6 min, an optimal configuration emerged, in which the front side showed strong gel penetration, while the back side remained fiber‐dominated, achieving the best deformation matching between gel and fibers. By contrast, 8 min spinning produced excessively dense fiber networks, impeding gel infiltration and creating structural defects that limited deformability [40]. Its non‐monotonic evolution reflects the transition from a “gel‐dominated” to a “fiber‐dominated” state, with 6 min of spinning achieving the optimal balance between hydrogel infiltration and fiber reinforcement.

Since the gel tends to infiltrate the fiber network to some extent after spin‐coating, making its exact amount difficult to quantify. We fixed the fiber thickness by spinning for 6 min and then studied the PU‐GBPC membranes as a function of the fiber weight fraction (Figure 2c; Figure S10). The fiber weight fraction can be calculated by weighing the fibers before and after spin‐coating. As the fiber fraction increased (and the gel fraction correspondingly decreased), the stronger confinement imposed by the PU fiber network gradually enhances the tensile strength and toughness of the material. At a fiber fraction of 70%, the PU‐GBPC exhibits a toughness of 55.62 MJ m^−3^, an elastic modulus of 0.07 MPa, and a stress of 24.87 MPa (Figure 2d; Figure S11), and can support the weight of a 400 g water bottle (Figure S12). At this thickness, the hydrogel almost completely filled the fiber gaps, producing a stiffer, fiber‐constrained composite with reduced elongation at break (Figure S13). Conversely, when the fiber content decreases to 30%, the excessive hydrogel volume causes gel‐fiber decoupling, weakening the reinforcing effect and reducing the elongation at break (Figure S14) [41]. These results highlight that an optimal balance between fiber density and hydrogel thickness is critical for achieving superior mechanical performance. Specifically, when the PU fibers were spun for 6 min and the subsequently spin‐coated hydrogel constituted 70% of the total, the most favorable fiber‐gel interpenetration was achieved, thereby endowing the composite with exceptional toughness [40].

Moreover, under the vertical puncture of sharp objects, PU‐GBPC can inhibit the initiation and propagation of cracks caused by stress concentration, showing excellent puncture resistance (Figure 2e). We further investigated the effect of DES on the tensile properties of PEDOT:PSS hydrogels. As shown in Figure 2f,g, DES doping significantly enhanced flexibility and ductility by reducing the rigidity of the PEDOT:PSS matrix. Mechanistically, DES reduces Coulombic repulsion between negatively charged PSS chains and partially reorganizes rigid PSS‐enriched domains. Simultaneously, DES molecules intercalate into the polymer network, solvating and mobilizing the polymer chains. This microstructural reorganization endows PEDOT:PSS composite hydrogels with markedly improved stretchability and resilience (Figure 2h).

To assess fatigue resistance, cyclic tensile tests were conducted under 100–500% strain (Figure S15a, b). Hysteresis was initially pronounced but diminished sharply after the first cycle, indicating stress‐induced structural rearrangements. When stretched to 200% strain and rested for 20–60 min, reduced hysteresis was observed, consistent with irreversible fiber sliding and network reorganization into a new stable architecture (Figure S16). Under 100% strain for 500 cycles, hysteresis stabilized at ≈45% after the first cycle, with subsequent loops nearly overlapping (Figure S17a, b). This behavior mirrors classical double‐network hydrogels, in which initial loading irreversibly damages the brittle network, while subsequent cycles yield an elastic, resilient response [42, 43]. Finally, during repeated balloon inflation‐deflation, the membranes withstood large reversible deformation without fracture, highlighting robust mechanical integrity and operational repeatability (Figure S18).

### 2.3 The Janus Property of PU‐GBPC Hydrogel Sensors

**FIGURE 3:**
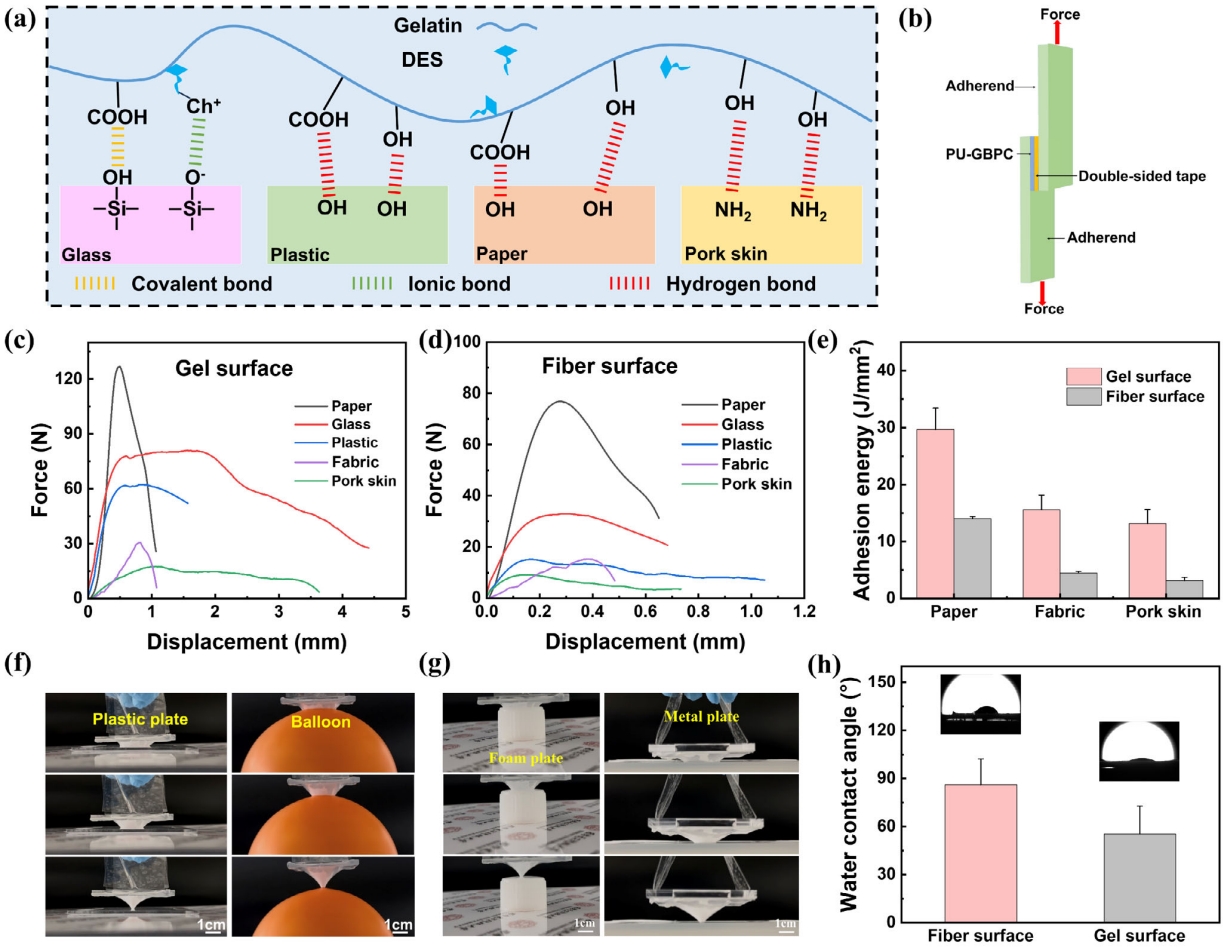
The Janus Property of PU‐GBPC Hydrogel Sensors. a) Schematic diagram of the adhesion mechanism of the PU‐GBPC hydrogel sensor. b) Schematic diagram of lap shear for adhesion testing. c) Displacement and force distribution of the gel surface in lap shear experiments on different substrates. d) Displacement and force distribution of the fiber surface in lap shear experiments on different substrates. e) Asymmetric adhesion ability of PU‐GBPC on filter fabric, paper and pork skin. f, g) The fiber surface of the PU‐GBPC hydrogel sensor is adhered to the plastic plate, and the peeling process of the gel surface on the plastic plate, balloon, foam plate and metal plate. h) Water contact angles on both sides of the PU‐GBPC hydrogel sensor.

Adhesive performance is pivotal to the reliability of the PU‐GBPC composite ultrathin hydrogel sensor, as it minimizes signal artifacts from interfacial slippage and ensures conformal contact with dynamic and irregular human skin surfaces [44]. The asymmetric adhesion of the PU‐GBPC hydrogel membrane's Janus structure stems from the spatially asymmetric distribution of microscopic mechanical interlocking and chemical bonding, enabled by precisely controlled partial fiber penetration. On the strongly adhesive hydrogel side, gelatin molecules provide abundant hydrogen bonding sites, forming a 3D hydrogen bond network (Figure 3a). Dynamic coordination between B(OH)_4_^−^ ions and surface hydroxyl groups further enables self‐regulating adhesion, while DES temporarily softens the interface to facilitate polymer chain diffusion and entanglement, thereby enhancing adhesion [45, 46]. During peeling, the mechanical force from the robust 3D interpenetrating network of the hydrogel and partial fibers, coupled with the synergistic effects of abundant hydrogen bonds and van der Waals forces at the interface, yields high peel strength and tough fracture. Figure S19 shows the hydrogel side bears 50 g or even 100 g without peeling. In contrast, the fiber side's adhesion depends only on weak van der Waals forces, enabling easy, low‐energy peeling. Video S2 shows the fiber side's adhesion to the metal substrate is significantly weaker than the hydrogel side's.

To further clarify the adhesion difference between the two sides of the composite hydrogel membrane, researchers fixed one side of the membrane with double‐sided tape and performed lap shear tests on the other side (Figure 3b). As presented in Figure 3c,d, the adhesion energies of the gel side to plastic, paper, glass, fabric, and pigskin were 39.19 J mm^−2^, 29.64 J mm^−2^, 20.21 J mm^−2^, 15.58 J mm^−2^, and 13.18 J mm^−2^, respectively‐far higher than those of the fiber side. On filter paper and pigskin substrates, the adhesion energy of the gel side was even more than twice that of the fiber side (Figure 3e). Additionally, the gel side could adhere to various substrates such as glass, foam, and metal plates (Figure S20). To verify the asymmetric adhesion property, researchers fixed the edges of PU‐GBPC to a square plastic plate using biological tape, and then attached the gel side to plastic plates, balloons, metal plates, and foam plates (Figure 3f,g). It was observed that during peeling, the gel side exhibited good adhesion to different substrates, while the fiber side showed weak adhesion a characteristic that enables it to effectively avoid external interference when applied to human skin. Furthermore, water contact angle (WCA) tests (Figure 3h) revealed that the gel side had strong hydrophilicity (WCA ≈ 43°), which is attributed to the abundant polar groups in gelatin that form hydrogen bonds with water molecules. The WCA of the fiber side increased to 67.4°, indicating that the higher PU fiber content enhanced interfacial hydrophobicity. This asymmetric surface property ensures the stability of long‐term human skin signal monitoring [47].

### 2.4 Biocompatibility and Recyclability of PU‐GBPC Hydrogel Sensors

**FIGURE 4:**
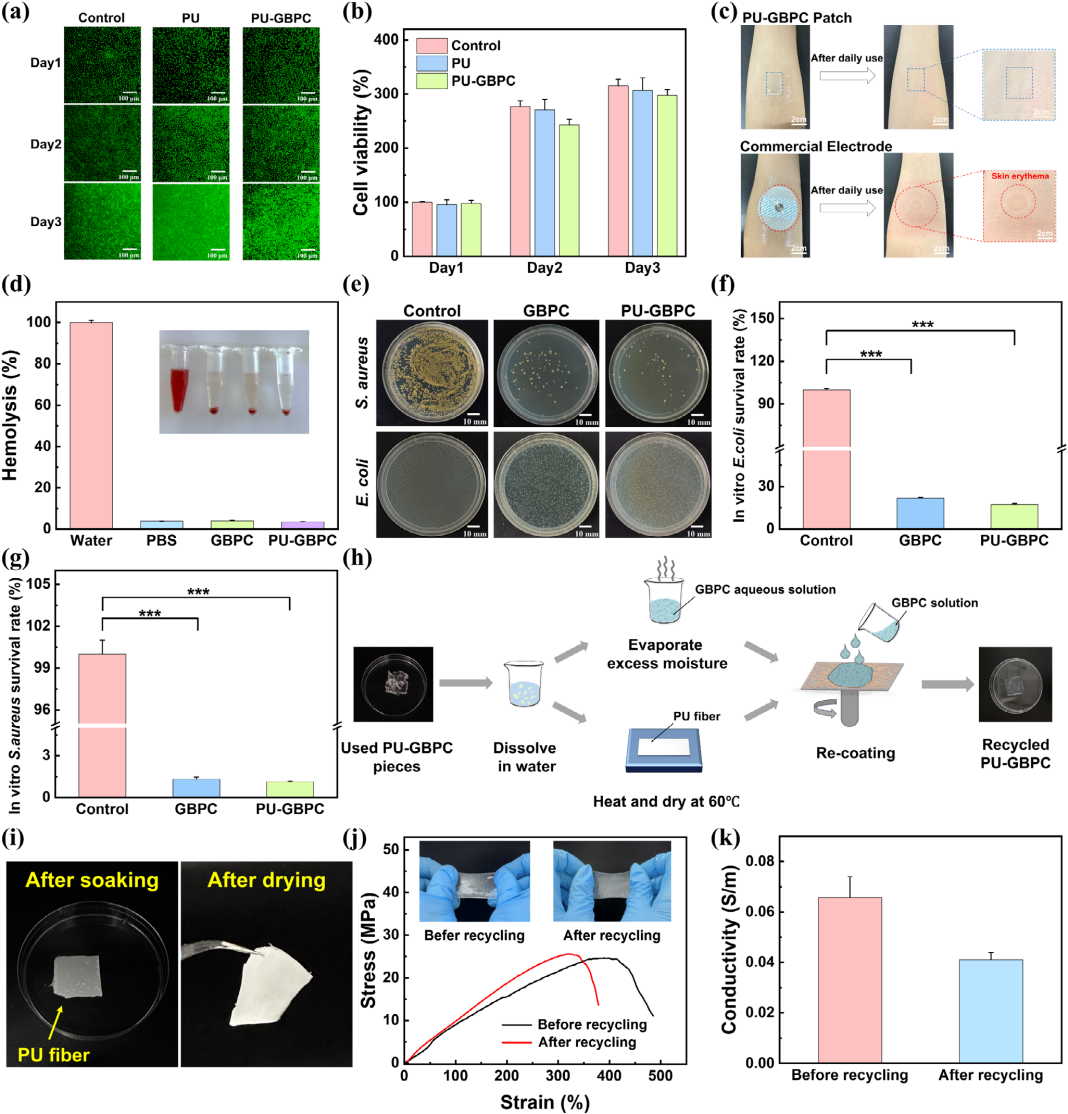
Biocompatibility and recyclability of PU‐GBPC hydrogel sensors. a) Live/dead staining images of the PU‐GBPC hydrogel co‐cultured with L929 cells for 24 h, 48 h, and 72 h. b) Cell viability of the PU‐GBPC hydrogel co‐cultured with L929 cells for 24 h, 48 h, and 72 h. c) Skin conditions after 24‐hour continuous wearing of the PU‐GBPC hydrogel sensor and commercial electrode sheets. d) The hemolysis rate of the PU‐GBPC hydrogel sensor. e) Morphology of Escherichia coli and Staphylococcus aureus after 12 h of co‐cultivation with GBPC and PU‐GBPC hydrogel sensors. f) Survival rate of *E. coli* after 12 h of co‐cultivation with GBPC and PU‐GBPC hydrogel sensors. g) Survival rate of *S. aureus* after 12 h of co‐cultivation with GBPC and PU‐GBPC hydrogel sensors. n = 3, *p < 0.05, **p < 0.01 and ***p < 0.001. h) Schematic diagram of the recycling process for PU‐GBPC hydrogel. i) The appearance of recycled PU fibers after soaking and drying. j) Comparison of mechanical properties of PU‐GBPC before and after recycling. k) Comparison of Conductive Properties of PU‐GBPC before and After Recycling.

To ensure the biosafety of the PU‐GBPC hydrogel sensor for wearable applications, the material components must be non‐toxic, stable, and non‐allergenic. Studies have shown that in DES with ChCl as the hydrogen bond acceptor (HBA), those using alcohols as hydrogen bond donor (HBD) exhibit significantly low cytotoxicity [48]. While glycerol, a generally recognized non‐toxic alcoholic substance, forms DES with ChCl that possess extremely low toxicity [49]. The cytocompatibility of pure PU fibers and PU‐GBPC hydrogels was assessed using live/dead staining and MTT assays. After 72 h of co‐culture, both groups showed cell viabilities exceeding 90% (Figure 4a,b; Figure S21). To further evaluate skin compatibility, PU‐GBPC films were worn on the forearm for 24 h, and even to the abdomen for 72 h. No erythema, irritation, or discomfort was observed, and the ultra‐thin films could be easily peeled off without causing inflammation (Figure 4c; Figure S22). In addition, hemolysis assays revealed a hemolysis rate of 3.5% (Figure 4d), well below the accepted threshold. These results collectively demonstrate excellent biocompatibility and safety for long‐term epidermal use. Beyond biocompatibility, antibacterial activity is essential to mitigate infection risks. Representative Gram‐negative (*E. coli*) and Gram‐positive (*S. aureus*) bacteria were cultured with PU‐GBPC hydrogels (Figure 4e). The survival rate of E. coli was reduced to 17% (Figure 4f), while S. aureus was nearly eradicated, with only 1.1% survival (Figure 4g). These results highlight potent broad‐spectrum antibacterial efficacy, ensuring cleanliness during prolonged wear [50].

Importantly, the PU‐GBPC hydrogel sensor also exhibits closed‐loop recyclability. The triple‐helical structure of gelatin enables thermoresponsive gel‐sol transitions, while reversible borate ester bonds allow water‐triggered disassembly (Figure 4h). The composite hydrogel dissolves after 1 h of immersion in 60°C water. Hydration primarily disrupts temporary interchain physical crosslinks (e.g., hydrogen bonds, ionic coordination, hydrophobic associations) via selective and reversible cleavage, without permanent breakage of polymer backbone covalent bonds or irreversible molecular degradation. Subsequent heating to evaporate the added water drives the GBPC hydrogel precursor solution to undergo thermodynamic‐driven rearrangement via Brownian motion. Re‐spin‐coating onto recycled, dried, and reused PU fibers yields new PU‐GBPC hydrogels (Figure 4i; Video S3). In this process, no material loss or gain occurs, yielding a physical crosslinking network highly similar to the original. This precise reconstruction stems from the unchanged primary structure of the polymer and intrinsic intermolecular interactions during cycling. Mechanical tests confirm complete retention of tensile strength after recycling (Figure 4j; Figure S23), while conductivity decreases slightly only due to partial reorganization of the dynamic conductive network (Figure 4k). Additionally, mechanical and electrical stability were tested at 30% and 70% cyclic humidity to ensure the composite hydrogel membrane remains insoluble at room temperature (Figure S24). This simple water‐based method eliminates secondary waste generation. The water‐soluble recycling process of PU‐GBPC provides a sustainable solution for recyclable electronic skins, significantly reducing electronic waste and environmental burdens from disposable flexible electronics.

To comprehensively evaluate the reliability of the PU‐GBPC hydrogel sensor in real‐world application scenarios, the team simulated various inevitable environments during long‐term wear. These include exposure to sweat‐secreting skin surfaces for 48 h, 10 humidity cycles between 30% and 70%, 100 friction cycles under 30% strain, and 100 human skin adhesion‐detachment cycles. Subsequently, the core electrical and mechanical properties of the sensor were systematically monitored (Figure S25). The results show that the sensor's toughness retained over 90% of its original value after the above four tests. Furthermore, the conductivity of the sensor increased by approximately 8% and 17% when exposed to skin sweat and high humidity environments, respectively. This is mainly attributed to the fact that water molecules significantly enhance the mobility of polymer segments, thereby improving the intrinsic ionic mobility within the hydrogel. Ions gain wider channels and lower movement resistance in the swollen network, which optimizes ionic transport efficiency [51]. This effect is far greater than the positive contribution of increased ions in sweat to conductivity. In conclusion, the PU‐GBPC hydrogel sensor provides solid experimental evidence and performance guarantees for its practical application in fields such as mobile health, personalized medicine, and human‐computer interaction that require long‐term, continuous, and reliable monitoring.

### 2.5 Temperature Sensing Performance of PU‐GBPC Hydrogel Sensors

**FIGURE 5:**
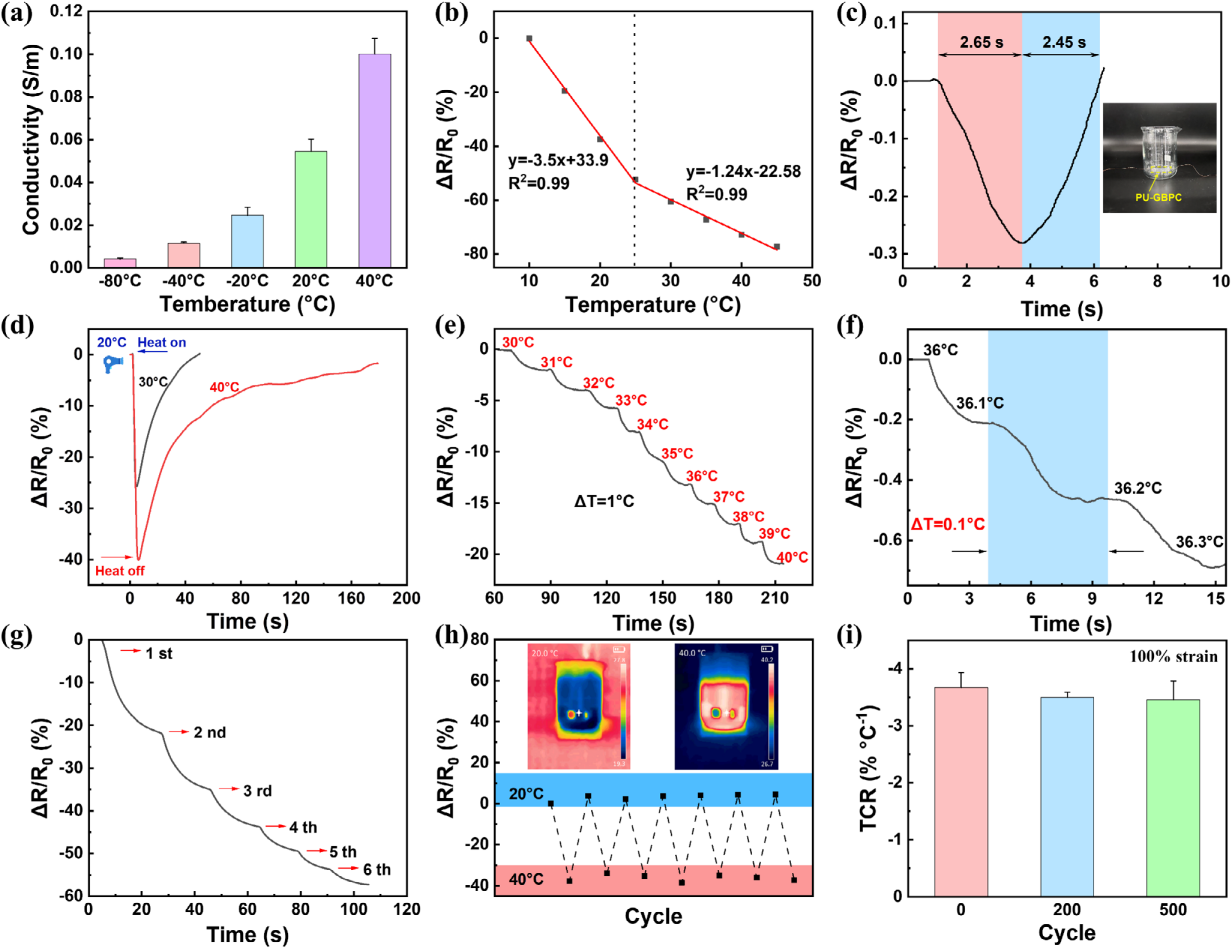
Temperature sensing performance of the PU‐GBPC hydrogel sensor. a) Changes in electrical conductivity at different temperatures. b) Negative resistance changes relative to temperature. c) Thermal response recovery time of the PU‐GBPC hydrogel sensor in contact with a heat source, with the inset showing the PU‐GBPC adhered to the wall of a beaker. d) High‐fidelity thermal sensing test using a hair dryer as the heat source at different temperatures. e) *∆R/R_0_* of the PU‐GBPC hydrogel sensor with continuous subtle temperature changes (∆T = 1°C). f) High‐resolution (∆T = 0.1°C) detection within the range of human body temperature monitoring. g) Resistance response curve during continuous filling with hot water. h) Stable and repeatable temperature response (10 cycles, 20–40°C), with the inset showing infrared thermal images of the PU‐GBPC hydrogel at 20°C and 40°C. i) TCR (Temperature Coefficient of Resistance) after 0, 200, and 500 cycles under 100% strain.

In the network of the PU‐GBPC hydrogel sensor, glycerol forms abundant antifreeze hydrogen bonds with water molecules, thereby locking free water and preventing the film from crystallizing at low temperatures [52]. As shown in Video S4, the PU‐GBPC hydrogel sensor still maintains good stretchability at ‐80°C. The ion‐electron dual‐conductive network in PU‐GBPC enables the composite hydrogel sensor to have a conductivity of 0.06 S m^−1^ at room temperature (Figure 5a). The conductivity varies systematically with temperature, reflecting a pronounced resistance response. Mechanistically, as temperature increases, the insulating PSS domains shrink, reducing the distance between adjacent PEDOT chains and thereby enhancing electron hopping [53]. Consequently, PU‐GBPC hydrogel sensor exhibits a negative temperature coefficient of resistance (TCR) (Figure 5b). Within 10–25°C, the TCR reaches 3.5% °C^−1^, while in 25–45°C it remains 1.24% °C^−1^, both with excellent linearity (R^2^ = 0.99). To probe thermal responsiveness, the hydrogel sensor was laminated onto a beaker wall, and water temperature was varied in real time. The hydrogel sensor demonstrated a rapid thermal response of 2.65 s (Figure 5c). When the hair dryer heats the hydrogel sensor in the Petri dish from the outside, the sensor can accurately distinguish between the two hair dryer models set at 30°C and 40°C. (Figure 5d).

Notably, the response times were identical across different inputs, underscoring high sensitivity and reproducibility. When the temperature changes are ∆T = 1°C (30‐40°C) and ∆T = 0.1°C (36‐36.3°C), it shows good temperature response and stability, indicating that the composite hydrogel sensor has high resolution (Figure 5e,f). Furthermore, transient temperature monitoring experiments, achieved by sequentially injecting isothermal hot water (80°C), revealed that the sensor can precisely capture rapid thermal fluctuations (Figure 5g). Long‐term stability was confirmed by cyclic tests, Figure 5h shows that when the temperature of the composite hydrogel sensor is cyclically changed for 10 cycles within the range of human body temperature (20–40°C). it can still maintain almost the same resistance signal. This indicates that the composite hydrogel sensor can maintain ideal thermal sensing performance for signal acquisition during continuous operation. The inset shows the temperature change of the gel under an infrared thermal imager. Moreover, after being stretched to 100% for 200 and 500 cycles, the TCR of the composite hydrogel sensor remains almost the same as that before stretching without obvious attenuation (Figure 5i), demonstrating exceptional mechanical robustness and reliable thermal sensing under dynamic deformation. Collectively, these results confirm that the PU‐GBPC hydrogel sensor delivers high sensitivity, rapid response, outstanding stability, and strong anti‐interference capability across physiologically relevant conditions. Such robustness ensures reliable temperature detection in dynamic environments and provides a solid foundation for the accurate fusion of multimodal health‐monitoring data.

### 2.6 Strain Sensing Performance of PU‐GBPC Hydrogel Sensors

**FIGURE 6:**
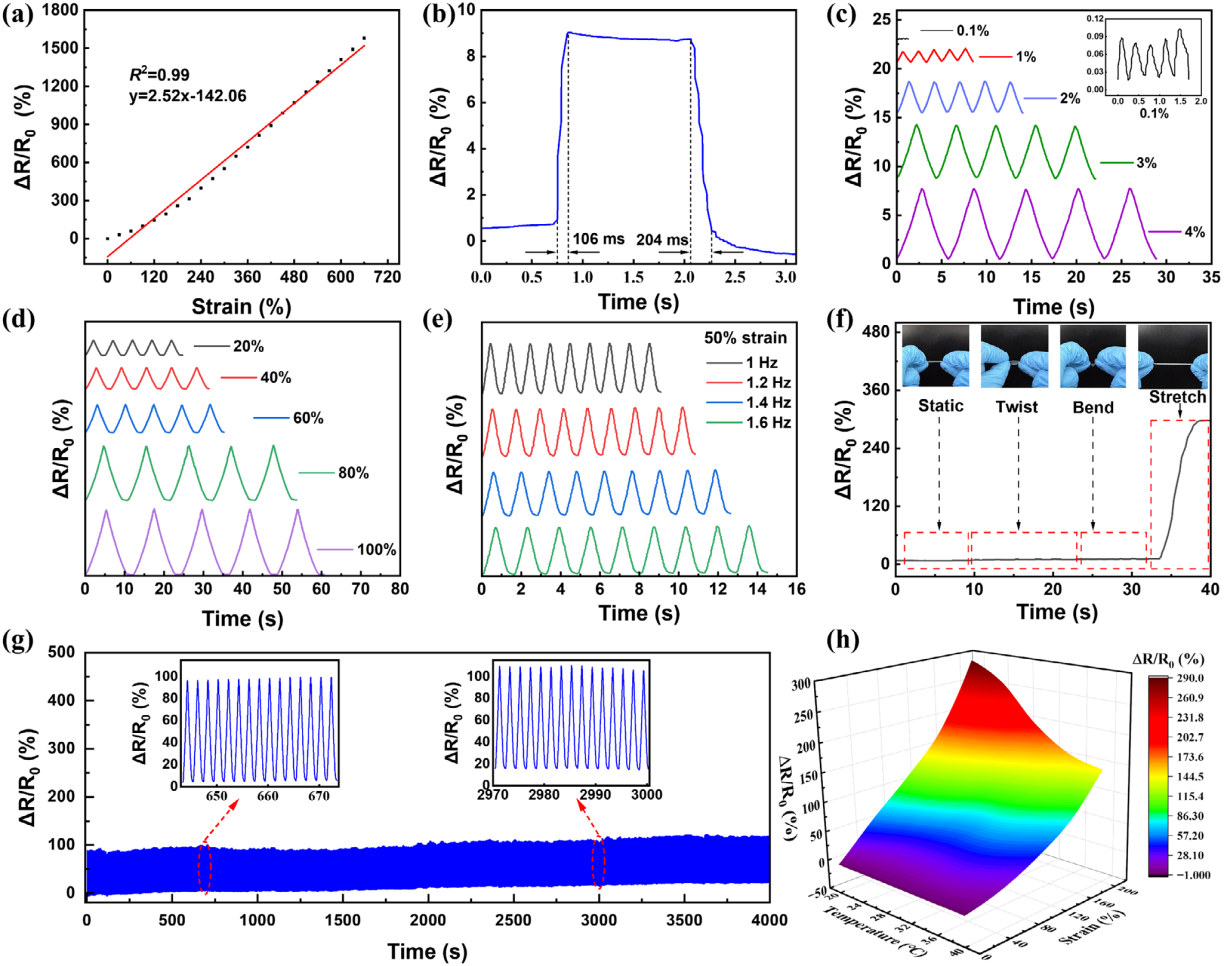
Strain sensing performance of the PU‐GBPC hydrogel sensor. a) Corresponding relationship between relative resistance amplitude and strain. b) Response recovery time to strain occurrence. c) Resistance change rate from 0.1% to 4% strain. d) Resistance change rate at 20–100% strain. e) Corresponding relationship between relative resistance amplitude and strain. f) Relative resistance change rates under stability, torsion, bending, and stretching conditions. g) Real‐time resistance changes during 2000 cycles of 50% stretching. h) *∆R/R_0_* at 20°C to 40°C and 200% tensile strain.

The ultra‐thin composite hydrogel membrane reinforced with PU fibers exhibits remarkable mechanical robustness and was integrated into flexible strain sensors. The PU‐GBPC hydrogel sensor demonstrates rapid recovery after stretching and maintains exceptional electrical stability. The sensing performance of the PU‐GBPC hydrogel sensor was evaluated by recording *∆R/R_0_* during tensile deformation. Owing to its ultra‐thin architecture, out‐of‐plane deformation is effectively suppressed, enabling a gauge factor (GF) of 2.52 over strains up to 600% with excellent linearity (R^2^ = 0.99, Figure 6a). This result highlights the high sensitivity and broad sensing range of the PU‐GBPC sensor. The response time under tensile loading and release is as short as 106 ms and 204 ms, respectively (Figure 6b). The sensor resolves minute strains (0.1% – −4%) with stable signals and shows negligible attenuation during cyclic loading‐unloading tests (Figure 6c). Moreover, progressive strain levels from 20% to 100% are reliably captured with preserved stability and linearity (Figure 6d). Stable responses are also maintained at different actuation frequencies during 10 repetitive cycles at 50% strain (Figure 6e). Importantly, the sensor exhibits high selectivity to tensile strain: torsional or bending deformations induce negligible resistance variations (Figure 6f), thereby minimizing interference from non‐stretching modes. The PU‐GBPC hydrogel sensor further demonstrates long‐term durability, with stable responses after 2000 consecutive cycles at 50% strain (Figure 6g). In addition, within the 200% strain range, the strain decreases to a certain extent with the increase of temperature, but the influence is very small within the body temperature range of 20–40°C (Figure 6h; Figure S26).

### 2.7 Application of PU‐GBPC Hydrogel Sensors in Multi‐Scale Force Monitoring

**FIGURE 7:**
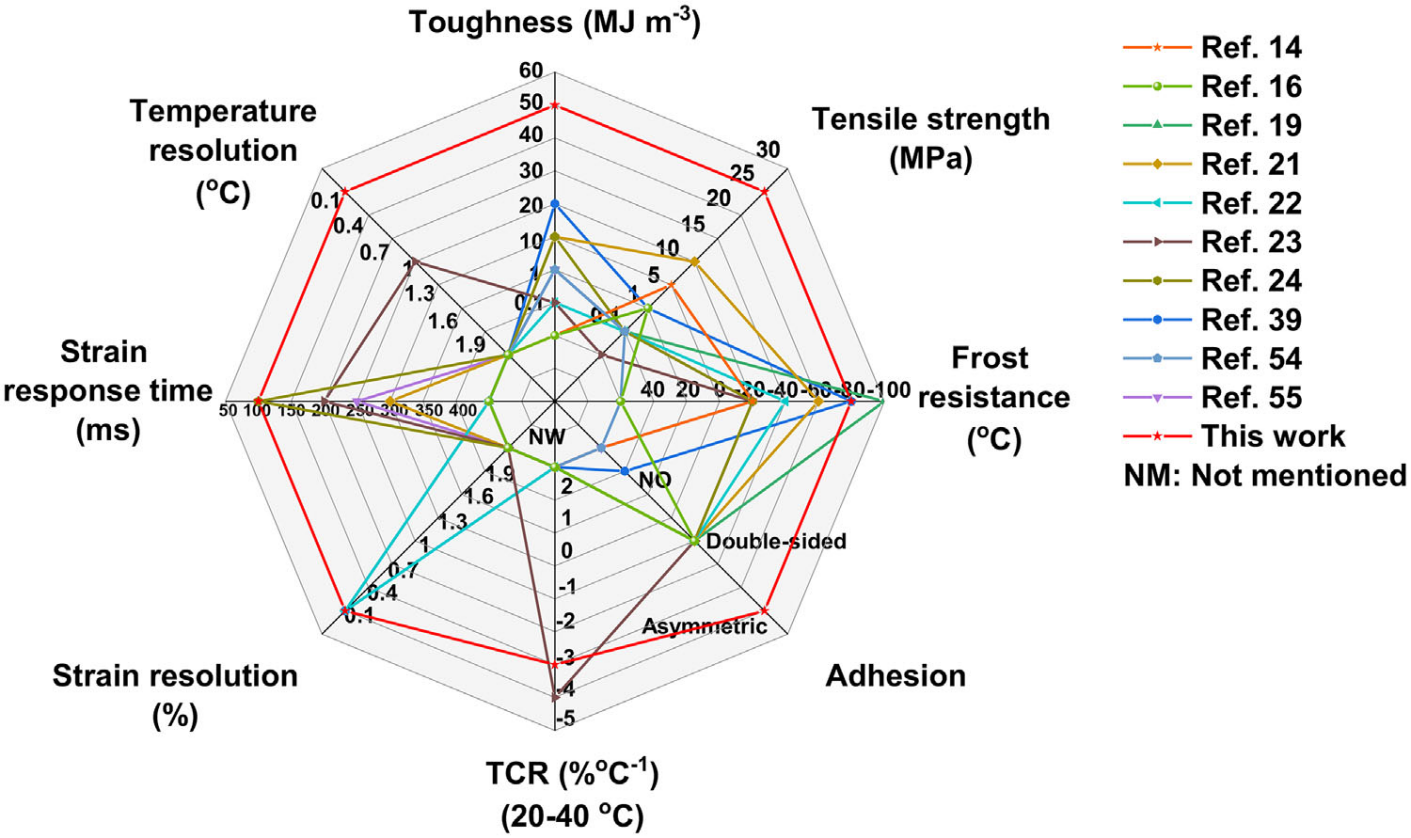
Comparison of the collective advantages of the PU‐GBPC hydrogel sensor with other hydrogel sensors reported in the literature.

Compared with recently reported conductive hydrogels, the core advantage of the PU‐GBPC hydrogel sensor lies in its ultra‐thin structure and high toughness, which facilitate superior skin adhesion and conformal contact on curved surfaces, thereby enabling more precise measurement of privacy‐sensitive physiological signals. Additionally, this hydrogel sensor exhibits excellent tensile strength, thermal sensitivity, low strain detection limit, rapid response time, asymmetric interfacial adhesion, freezing resistance, recyclability, water retention capacity, visual transparency, antibacterial properties, and biocompatibility [14, 16, 19, 21, 22, 23, 24, 39, 54, 55]. As illustrated in Figure 7 and Table S2, these characteristics make it an ideal choice for human healthcare, autonomous motion monitoring, and various biomedical applications.

**FIGURE 8:**
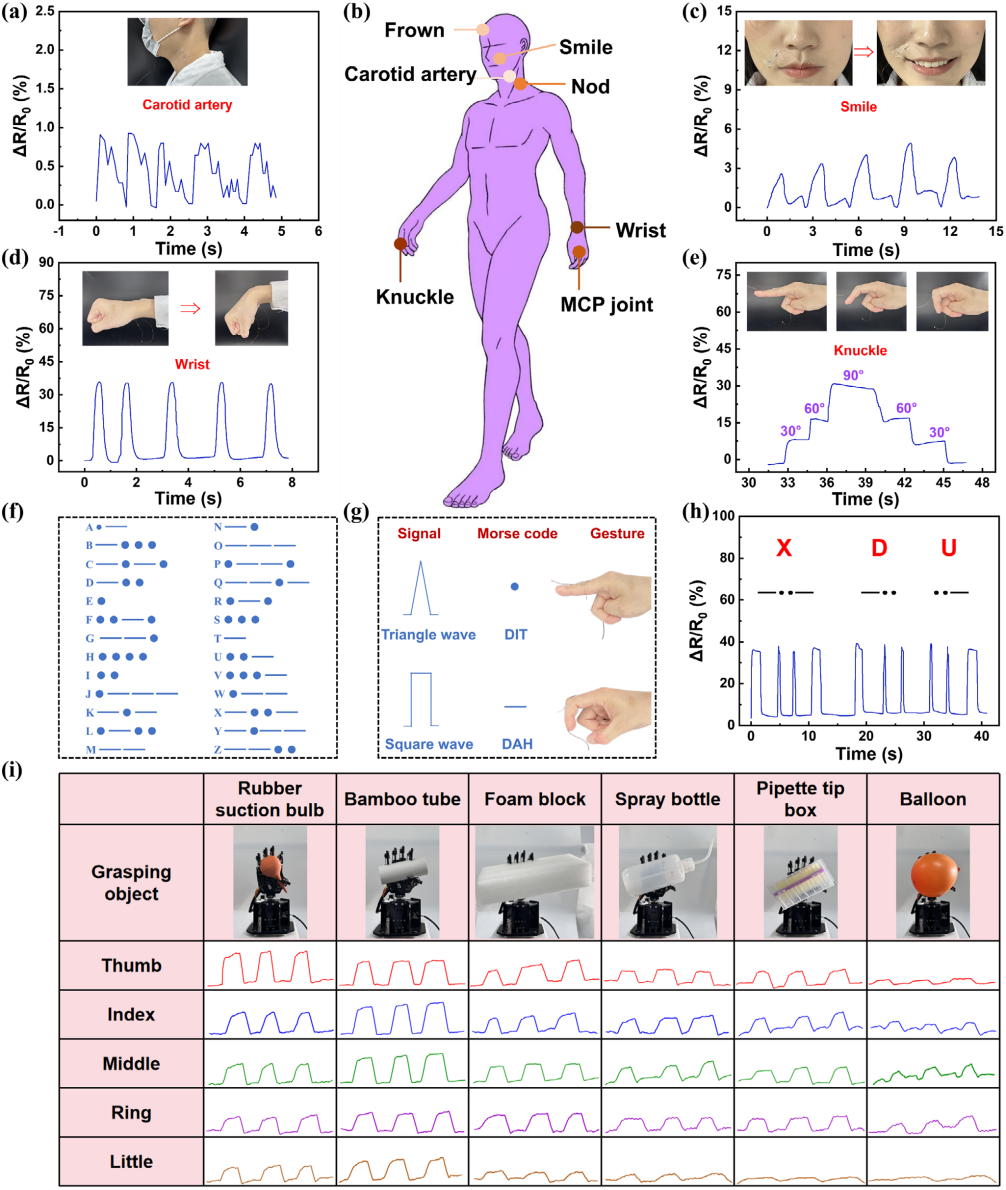
Signal changes of the PU‐GBPC hydrogel sensor in different application scenarios. a) Carotid artery signals detected by the PU‐GBPC hydrogel sensor. b) Electrical signals from different parts of the human body. c) Electrical signals of facial muscles when smiling. d, e) Electrical signals of the wrist joint and finger joints. f) Morse code table for 26 letters. g) Schematic diagram of signal codes. h) Electrical signals corresponding to “XDU”. i) Signal changes of the five fingers of the manipulator when grasping objects of different shapes in a low‐temperature environment.

Human skin endures strains of various intensities, ranging from subtle vibrational strains like pulses to more significant ones such as joint movements (Figure 8b). The PU‐GBPC hydrogel sensor can detect the full range of these strains and, with its high sensitivity, capture weak signals like human carotid artery pulses (Figure 8a). As shown in Figure 8c and Figure S27, the sensor can accurately detect subtle muscle strains associated with facial expressions such as frowning and smiling. When attached to the neck, it can measure the angle changes corresponding to nodding and head turning (Figure S28a, b). Upon hand attachment, it rapidly detects the bending degrees of metacarpophalangeal, finger, and wrist joints accurately, with resistance change rate dependent on bending degree (Figure 8d,e; Figure S29). When the finger joints are bent at 30°, 60°, and 90° respectively, the experimental results also confirm the excellent sensitivity of the PU‐GBPC hydrogel sensor. In Figure S30a, PU‐GBPC hydrogel sensors are installed at the joints of the five fingers, enabling sign language recognition (Figure S30b illustrates the sign language meaning of “in sequence”). The sensor transforms joint bending into high signal‐to‐noise ratio strain signals, delivering a concealed, biocompatible, safe and reliable option for information encryption. By arranging dots and lines to represent English letters (Figure 8f), short curves produce sharp peaks (corresponding to the Morse code ‘dot’), while long curves produce trapezoidal peaks (corresponding to the ‘line’) (Figure 8g). ‘XDU,’ ‘LOVE,’ ‘SOS,’ and ‘HI’ can all be represented by corresponding electrical signals (Figure 8h; Figure S31a–c). This demonstrates that PU‐GBPC sensors can support encrypted information transmission, significantly expanding their application potential.

Furthermore, due to its excellent sensing robustness at low temperatures, the PU‐GBPC hydrogel sensor can be used for mechanical gesture recognition and object grasping in harsh environments. We mounted an array of hydrogel sensors on the back of the fingers of a robotic arm and placed it in a low‐temperature environment to monitor its bending state at low temperatures. A multi‐channel system is used to monitor strain data in real‐time without crosstalk, and the data is transmitted to a smartphone via Bluetooth (Figure S32). When grasping objects of different shapes, the resistance change rate in each channel correlates linearly with finger bending (Figure 8i). Complex gestures, such as letters A‐F and numbers 1–6, produce distinctive, reproducible resistance patterns across the five fingers (Figure S33a,b). Collectively, these results validate that the PU‐GBPC hydrogel sensor meets the reliability and versatility requirements for human–machine interaction and gesture recognition when operating in specialized scenarios such as polar scientific research and cold‐chain logistics.

### 2.8 Application of PU‐GBPC Hydrogel Sensors in Gender‐Specific Physiological Function Monitoring

**FIGURE 9:**
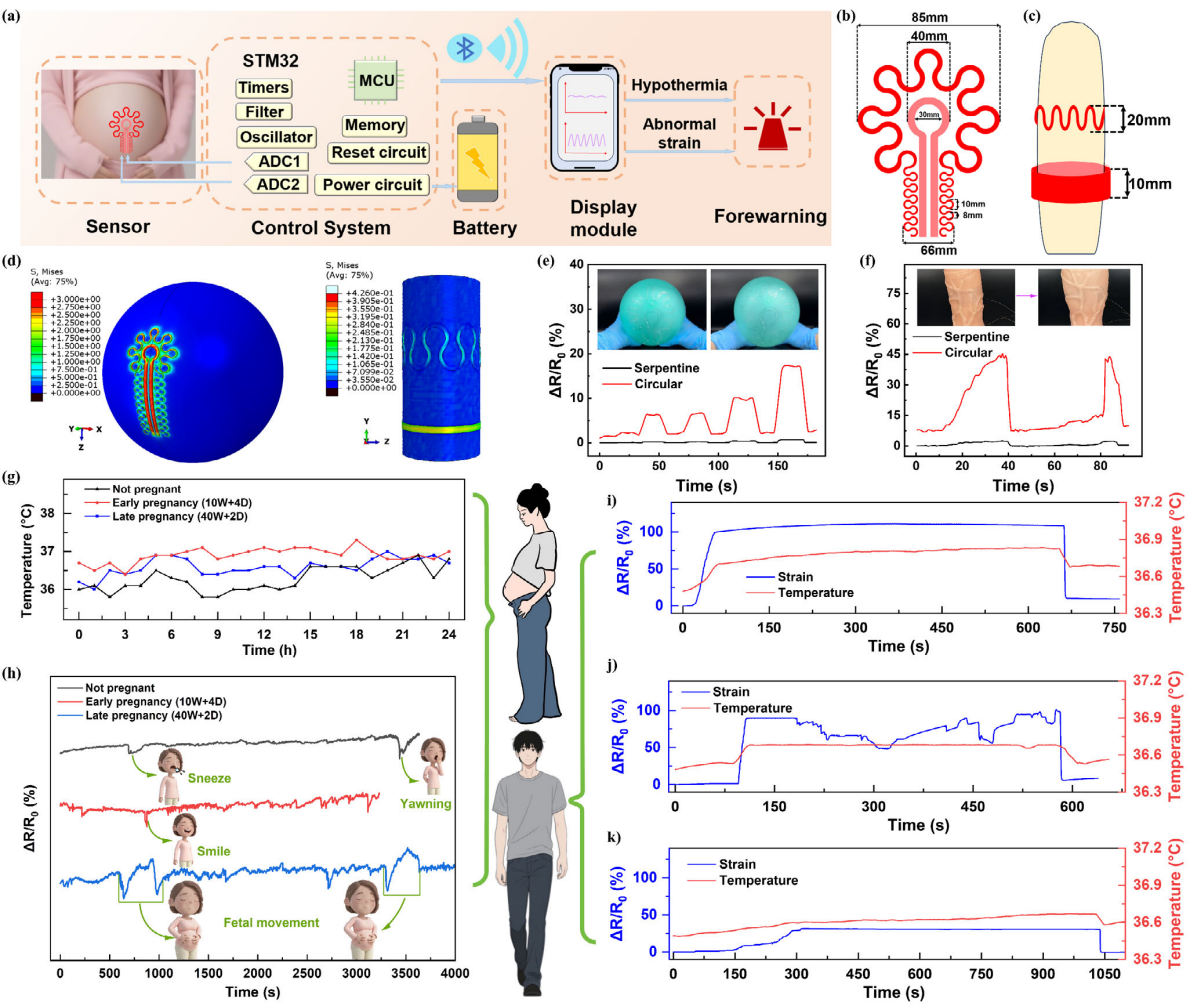
Application of the PU‐GBPC hydrogel sensor in gender‐specific physiological function monitoring. a) Schematic diagram of the equivalent circuit of the intelligent gender‐specific physiological function monitoring system. b) Serpentine ring structure for the abdomen of pregnant women. c) Ring structure for monitoring penile erection status. d) Finite element models comparing the stress distribution of serpentine sensors and ring sensors on spheres and cylinders. e) Signal comparison between serpentine sensors and annular sensors during simulated fetal movement. f) Signal comparison between serpentine sensors and annular sensors during simulated erection. g) 24‐hour abdominal temperature changes in volunteers who are non‐pregnant, in early pregnancy (10 weeks + 4 days), and in late pregnancy (40 weeks + 2 days). h) Continuous 1‐hour abdominal strain signals of non‐pregnant, early pregnancy, and late pregnancy volunteers. i–k) The strain and temperature signals of the penis during an erection in patients with normal, psychogenic, and organic ED.

To enable portable, home‐based self‐monitoring, the PU‐GBPC hydrogel sensor was integrated with a flexible printed circuit board (FPCB) incorporating Bluetooth, forming a dual‐mode signal acquisition system. Figure 9a illustrates the equivalent circuit of this system. Two independent analogue‐to‐digital conversion channels of the STM32 microcontroller acquire sensor signals, which are transmitted via Bluetooth to a mobile app for real‐time alerts of abnormal body temperature and strain, enabling closed‐loop physiological monitoring. Inspired by the structure of a scrunchie, the sensor comprises two functional components: a serpentine ring for temperature monitoring, minimizing interference from stretching, and an annular ring for strain detection. The core principle lies in leveraging the unique geometric configuration of this structure to physically establish mutually independent transmission paths and sensitive regions for the two signals. For pregnant women, a composite serpentine‐annular structure is employed, with the inner ring dedicated to strain monitoring and the outer ring to temperature measurement (Figure 9b). For male erectile function monitoring, an upper‐lower dual‐ring structure surrounding the penis is adopted (Figure 9c). Real‐time temperature feedback is used to dynamically calibrate strain signals, effectively eliminating thermal‐strain coupling (Figure 6h; Figure S26).

The effectiveness of the dual‐ring serpentine design was verified via finite element method (FEM) simulations using structural static analysis in Abaqus (Figure 9d). After fine mesh refinement and mesh independence validation, the simulations confirmed decoupling of strain and temperature. Videos S5 and S6 show that for 0%–15% strain in cylindrical and spherical molds, the annular structure stretches while the serpentine structure remains largely unchanged. When applied in the complete monitoring system, the electrical signal of the serpentine sensor is negligible relative to the annular sensor (Figure 9e,f), confirming successful decoupling and practical applicability for physiological monitoring. In addition, the signal baseline of the PU‐GBPC hydrogel sensor remains stable after 6 h of continuous operation (Figure S34). Such long‐term stability ensures the continuity and accuracy of data collection in the uncontrolled home environment, laying a data foundation for establishing personalized health baselines.

This system is ultimately used for intelligent gender‐specific physiological function monitoring. When the serpentine ring model is attached to the abdomen of a pregnant woman, it can simultaneously capture uterine contraction strain waveforms and local temperature signals, and after clinical validation, further enable dynamic evaluation of abnormal uterine activities during pregnancy. Figure 9g shows the 24‐hour abdominal temperature changes of volunteers who were non‐pregnant, in early pregnancy (10 weeks + 4 days), and in late pregnancy (40 weeks + 2 days). In early pregnancy (0–12 weeks), the basal body temperature is consistently 0.3–0.5°C higher than that before ovulation (non‐pregnant), which is a key indicator of stable embryo implantation (reflecting corpus luteum function). After 12 weeks, the fluctuation of body temperature tends to slow down; if the temperature rises abnormally (>37.2°C) during this period, it may indicate subclinical infection or abnormal thyroid function. In late pregnancy (28–40 weeks), with the increase of metabolic load, the basal body temperature rises physiologically by 0.1–0.2°C. However, if it persists at ≥37.8°C, high vigilance should be paid to chorioamnionitis or the onset of preterm contractions, especially when accompanied by decreased fetal movement, which suggests an increased risk of fetal distress in utero [56].

Figure 9h monitors the continuous 1‐hour abdominal strain signals of non‐pregnant, early‐pregnancy, and late‐pregnancy volunteers. The excellent sensitivity of the PU‐GBPC sensor detects varying degrees of abdominal changes in the subjects (such as sneezing, yawning, and laughing). These changes result from active muscle contractions that pull the abdominal wall inward, subjecting the sensor to a compressive strain. The characteristics of such signals‐sudden onset, high slope, and short duration‐are consistent with the explosive nature of muscle contractions. In contrast, fetal movements originate from the autonomous, bottom‐up physical activities of the fetus within the uterine cavity. Whether limb stretching, kicking, or whole‐body rolling, essentially the fetus pushes against the uterine wall, exerting an inward‐outward thrust on the maternal abdominal wall and inducing tensile strain in the attached sensor. More importantly, uterine contractions involve the regular, coordinated tightening of the entire uterine myometrium. Their signal characteristics are not transient sharp peaks but broad, mound‐shaped waves with a slow rise, a plateau phase, and a slow decline, lasting from tens of seconds to several minutes and exhibiting clear regularity and periodicity. Therefore, integrating signal direction (positive/negative), morphology (sharp/broad), duration (transient/sustained), and sequence pattern (isolated/regular) enables capture of genuine fetal activity and potential abnormal uterine contractions, offering high‐quality, low‐noise data for subsequent medical assessment [57]. Through continuous temperature‐uterine contraction dual‐modal monitoring, hypertensive disorders in pregnancy (abnormal elevation of morning temperature in late pregnancy) and occult infections (disappearance of circadian rhythm) are expected to be identified early, providing a critical time window for intervention.

The PU‐GBPC sensor can also monitor male penile erectile function. When the penis undergoes 40% deformation, the tension exerted by PU‐GBPC is only 1.43 N/cm^2^, ensuring safe and comfortable patient monitoring. As shown in Figure S35, volunteers wearing the dual‐ring system underwent a 10‐minute erectile process while temperature and strain signals were recorded under normal, psychological ED, and organic ED conditions. Normal erection is characterized by a sudden increase in radial strain, which is synchronized with the increase in skin temperature (Figure 9i). Psychological ED, due to excessive activation of the sympathetic nervous system, shows a leading surge in temperature with a lag in strain (Figure 9j). Organic ED, due to vascular endothelial dysfunction, exhibits a slow increase in strain, sometimes accompanied by abnormal temperature distribution (Figure 9k) [58]. Typing diagnosis is achieved through radial strain‐temperature collaborative analysis. However more rigorous clinical diagnostic conclusions require additional comparisons of clinical information from larger sample sizes and further clinical validation. The fully autonomous system integrated with an energy supply module may improve patient compliance, providing a new approach to home monitoring in privacy‐sensitive fields such as reproductive health. Ultimately, it is promising to enable a fundamental transformation in the “patient‐centered” intelligent health management model.

## 3 Conclusion

This study developed a clinically valuable ultra‐thin multimodal Janus hydrogel sensor toughened by PU fibers. Adopting a bionic dragonfly structure, its toughness is increased to 78 times that of pure hydrogel, enabling non‐invasive skin contact. Through the synergy of ion‐electron dual pathways, the sensor exhibits high strain sensitivity (2.52), rapid response, good stability (≥2000 cycles), and excellent linear temperature response regulated by PEDOT:PSS. It can be recycled through a simple heating process. Combined with FPCB, the study designed a dual‐ring serpentine structure for intelligent gender‐specific physiological function monitoring and early warning. Additionally, a physical‐algorithmic dual decoupling method for temperature‐strain signals was established, and this method has been verified through finite element simulations. The system can synchronously capture uterine contraction strain and temperature changes, and is anticipated to dual‐modal dynamic assessment of abnormal uterine activities during pregnancy. Meanwhile, as a monitoring ring for male penile erectile function, it shows great potential in distinguishing between psychogenic and organic ED through collaborative analysis of radial strain and temperature. This autonomous system, after clinical validation, is promising to improve patient compliance, enable home‐based privacy‐sensitive monitoring, and promote the transformation of medical monitoring from an “intermittent hospital‐centered” model to a “continuous home‐based personalized” model.

## 4 Experimental Section

### 4.1 Material

Thermoplastic polyurethane (TPU, 1185A) was supplied by BASF. Tetrahydrofuran (THF, 99.0%), N,N‐dimethylformamide (DMF, 99.5%), glycerol (Gly, 99%), choline chloride (ChCl, 98%), and sodium tetraborate decahydrate (Borax, 99.9%) were purchased from Macklin. Poly(3,4‐ethylenedioxythiophene)‐poly(styrene sulfonate) (PEDOT:PSS) was purchased from Aladdin. Type A gelatin powder (Bloom number: 240–300) was purchased from Shanghai Maokang Biotechnology Co., Ltd.

All reagents used in this work were analytical grade reagents and do not require further purification before use. The metal steel plates used for spinning were custom‐cut, with sizes including 2 cm × 2.5 cm, 3 cm × 3 cm, 5 cm × 8 cm, and 10 cm × 10 cm, purchased from Taizhou Mingju Metal Products Co., Ltd. Commercial therapeutic electrode patches were purchased from Nanjing Dalun Medical Technology Co., Ltd.

### 4.2 Preparation of Electrospun TPU Fibers

TPU (2 g) particles were dissolved in a mixed solution of THF (5 mL) and DMF (5 mL), stirred overnight, to obtain a 20 wt% TPU electrospinning solution. The electrospinning voltage was 16 kV, and the spinning rate was 0.003 mm s^−1^. TPU fibers were collected on a metal plate, with each fiber spun at the same position to improve the consistency of fiber thickness. The distance from the spinneret tip was 24 cm. To optimize the electrospinning thickness, pure PU spinning membranes with different electrospinning durations were prepared. The entire electrospinning process was conducted in a professional high‐voltage electrospinning machine (YFSP‐T, Tianjin Yunfan Technology Co., Ltd.).

### 4.3 Preparation of PU‐GBPC Hydrogel Sensors

First, glycerol (3.68 g) and ChCl (3.0 g) were heated and stirred using a water bath (DF‐101SR, Shanghai Xiniu Laibo Instrument Co., Ltd., China) at 60°C for 2 h to obtain DES. PEDOT:PSS (0.1 mL) was dissolved in deionized water (1.05 mL), and the two solutions were mixed and heated with stirring in a 60°C water bath. Finally, gelatin (1.15 g) and borax (0.69 g) were added, and the mixture was heated and stirred in a 60°C water bath to obtain a uniform hydrogel precursor solution.

Metal sheets with electrospun PU fibers were attached to a spin coater (EZ4‐S, Jiangsu Lebo Scientific Instrument Co., Ltd., China) or a blade coating machine (PF400‐H, Wuxi Yiri New Materials, China). The hydrogel precursor solution was quickly taken out of the water bath and dropped onto the PU fibers, followed by standing for 5 min to obtain an organic composite hydrogel (PU‐GBPC) where PU fibers were integrated with the hydrogel. Among them, the PU‐GBPC with a 70% fiber fraction was prepared by spin‐coating, with the speed and acceleration kept constant throughout the process. In contrast, blade coating could produce large‐area PU‐GBPC with uniform thickness. Similarly, PU‐GBPC hydrogel without glycerol was synthesized as a control. In addition, the hydrogel precursor solution was directly spin‐coated/doctor‐coated onto metal sheets to obtain pure hydrogel (GBPC) without PU fibers.

### 4.4 Characterization and Application of PU‐GBPC Hydrogel Sensors

#### 4.4.1 Morphological Characterization

The cross‐section of the PU fiber network and the two sides and cross‐section morphology of the composite organic hydrogel (PU‐GBPC) were observed using a field emission scanning electron microscope (SEM, Tescan Mira 4) at an acceleration voltage of 10 kV. Infrared spectroscopy was performed using a Fourier transform infrared spectrometer (FTIR) (Thermo Fisher Scientific Nicolet iS20). The samples were freeze‐dried, ground into powder, and scanned at room temperature in the range of 400–4000 cm^−1^.

#### 4.4.2 Mechanical Properties and Strain Sensing

Mechanical testing was conducted using a universal testing machine (Zwick/Roell) equipped with a 200‐Newton force sensor. Rectangular samples (25 mm × 20 mm) with different gel thicknesses but the same PU fiber thickness, and rectangular samples (25 mm × 20 mm) with the same gel thickness but different PU fiber thicknesses were subjected to uniaxial tensile testing. The maximum tensile stress and strain were determined at the point of fracture. The modulus of elasticity was calculated from the slope of the initial (10%–20%) region of the stress–strain curve. Toughness was determined by the area under the stress‐strain curve. Dissipated energy was calculated based on the enclosed area between the loading and unloading curves, and the dissipation ratio was defined as the ratio of energy dissipation after each cycle. To ensure reproducibility of results, we used stress‐strain curves from three parallel samples to calculate all parameters.

#### 4.4.3 Temperature Sensing

The temperature changes of the composite organic hydrogel sensor PU‐GBPC were monitored using an infrared thermal imager (UTi120S, Wuhan Lanmi Trading Co., Ltd., China). To accurately determine the temperature, experimental data were recorded after the fluctuation of the current curve stabilized.

#### 4.4.4 Moisture Retention

Glycerol hydrogel without PU fibers and pure water hydrogel were placed in an oven at 37°C (BCD‐156B, Hefei Rongshida Electrical Appliances Co., Ltd., China) for 30 days, and their weights were measured at the same time each day.

#### 4.4.5 Gas Permeability

Gas permeability was assessed using WVTR. The coverings were blank, cling film, PU fiber membrane (6 min), and PU‐GBPC composite hydrogel membrane, which were placed in glass bottles containing 10 mL of deionized water and placed in a constant temperature chamber at 37°C and 50% humidity. The test sample radius was 11.57 mm. The weight loss of each bottle was measured every hour for 12 h.

#### 4.4.6 Adhesion

A universal testing machine (Zwick/Roell) was used to perform the lap shear test for determining the adhesive strength. All the rectangular test samples had a size of 20 mm × 25 mm, and they were clamped between two substrates, where one side of the substrate was adhered to the non‐testing side of the sample with double‐sided tape. After preloading for 10 min, stretch the sample at a constant rate of 10 mm min^−1^ until it separates from the substrate. The adhesive strength was calculated as the maximum load divided by the initial bonding area. Additionally, the gel surface of the composite hydrogel membrane will be adhered to different substrates.

#### 4.4.7 Recyclability

Collect the prepared 3 cm × 3 cm PU‐GBPC composite hydrogel membrane in a 1 mL sample vial, add 0.5 mL of deionized water, let it sit for 1 h, remove the PU fiber membrane with tweezers, and dry it on a hot plate for about 1 min. The deionized water containing the sol was placed in a 60°C oven to evaporate the water, yielding the hydrogel precursor solution. Re‐coating this solution onto the PU fiber membrane yields a new PU‐GBPC.

#### 4.4.8 Cytotoxicity Testing

L929 cells were maintained in 1640 medium containing 10% (v/v) fetal bovine serum and 1% antibiotic‐antimycotic solution (100 U·mL^−1^ penicillin, 100 µg·mL^−1^ streptomycin) at 37°C under 5% CO_2_. Cells were seeded into 96‐well plates at a density of 1×105 cells.mL^−1^ (100 µL per well) and incubated at 37°C. After 24 h, the supernatant was discarded and replaced with hydrogel extract. Control wells received an equal volume of 1640 medium supplemented with 0.1% DMSO. Following incubation periods of 24, 48, or 72 h, MTT working solution (3‐(4,5‐dimethylthiazol‐2‐yl)‐2,5‐diphenyltetrazolium bromide; 10 µL per well) was added to each well, followed by 4 h of incubation. Five replicate wells were used per condition to ensure reproducibility and accuracy. Absorbance was subsequently measured at 450 nm using a microplate reader. Cell survival rate, calculated using Formula 4, served to assess cytotoxicity. Additionally, Live/Dead staining was performed on all groups, with the morphology and growth status of L929 cells exposed to gel extracts documented via fluorescence microscopy.

#### 4.4.9 Hemolysis Test

Five milliliters of fresh blood was centrifuged (2000 rpm, 5 min) and red blood cells (RBCs) collected. The RBCs were washed thrice with phosphate‐buffered saline (PBS) and resuspended in PBS to a final volume of 2 mL. Subsequently, 0.5 mL aliquots of the RBC suspension were combined with 0.5 mL PBS extracts from different hydrogel groups to constitute test groups. Controls included a PBS incubation group (negative control) and double‐distilled water (positive control). RBC suspensions were incubated with test and control solutions at 37°C (150 rpm, 30 min). Post‐incubation, samples were centrifuged (2000 rpm, 5 min) and supernatants collected. Absorbance of supernatants was measured at 570 nm using a microplate reader. Triplicate samples were analyzed per condition to ensure reproducibility and accuracy.

#### 4.4.10 Antimicrobial Properties

The antibacterial activity of the hydrogel sensors was evaluated using the liquid co‐culture method, with Escherichia coli (*E. coli*) and Staphylococcus aureus (*S. aureus*) selected for the assessment. 500 µL of bacterial suspension (either *E. coli* or *S. aureus*, OD600 = 0.5) was inoculated into 30 mL of sterile Luria‐Bertani liquid medium. GBPC and PU‐GBPC hydrogels (with a total volume of 200 µL, 100 mg·mL^−1^ for each) were added to the cultures. The co‐cultures were incubated at 37°C with a shaking speed of 120 rpm for 12 h. After incubation, the suspensions were serially diluted to 1×10^−3^ and inoculated onto the corresponding plates. After incubating for 16–18 h, the colony‐forming units were counted.

#### 4.4.11 Real‐Time Monitoring

A strain sensor array consisting of five hydrogel sensors was mounted on the back of the robot's fingers to detect the bending of each knuckle. Both ends were connected with copper wires and then fixed with bio‐tape to facilitate the transmission of electrical signals. The relative resistance changes of the five channels were respectively obtained through a commercial resistance‐capacitance tester (LZ‐01ARC (M), Linkzill, China). Similarly, the hydrogel sensor can also monitor human skin signals. With the consent of the volunteers, the sensor sensing performance test and specific physiological signal monitoring of their body movements were carried out. The volunteer's body experiments were conducted under from the Medical Ethics Committee of the Northwest University (250627095).

#### 4.4.12 Pregnancy Monitoring and Penile Erection Function Monitoring

A 10 cm × 10 cm PU‐GBPC hydrogel membrane was cut into ring‐shaped serpentine and rectangular shapes using a laser cutter (YFSP‐T, Yunfan (Tianjin) Instrument Co., Ltd., China), with both ends connected to a flexible circuit board via copper wires. The circuit board was placed in a pocket of clothing. During pregnancy monitoring, the serpentine ring‐shaped hydrogel sensor was positioned on the outer ring, and the ring‐shaped hydrogel sensor on the inner ring. To distinguish the impact of pregnancy on women's body temperature and abdominal strain, we selected 3 volunteers each from the non‐pregnant group, the first trimester group, and the third trimester group. For penile erection function monitoring, the serpentine ring‐shaped and ring‐shaped hydrogel sensors were sequentially wrapped and adhered to the penis. To distinguish the differences in temperature and penile changes during an erectile state between patients with different types of ED and non‐affected individuals, we selected 3 volunteers each from the normal group, the psychogenic ED group, and the organic ED group. The basic demographic characteristics and clinical features of the volunteers were shown in Table S3.

#### 4.4.13 Other Characterizations

Transmittance was measured using an enzyme‐linked immunosorbent assay (UV‐1750, Shimadzu) at 300 nm–800 nm to determine the light transmittance of pure hydrogel membranes (GBPC) and composite organic hydrogels (PU‐GBPC).

The water contact angle was the minimum sliding amount at a stretching rate of 200 mm min^−1^. Wettability was characterized using a dynamic contact angle measuring instrument (OCA20, Dataphysics).

## Conflicts of Interest

The authors declare no conflicts of interest.

## Supporting information




**Supporting File 1**: advs73478‐sup‐0001‐SuppMat.docx.

**Supporting File 2**: advs73478‐sup‐0002‐VideoS1.mp4.

**Supporting File 3**: advs73478‐sup‐0003‐VideoS2.mp4.

**Supporting File 4**: advs73478‐sup‐0004‐VideoS3.mp4.

**Supporting File 5**: advs73478‐sup‐0005‐VideoS4.mp4.

**Supporting File 6**: advs73478‐sup‐0006‐VideoS5.mp4.

**Supporting File 7**: advs73478‐sup‐0007‐VideoS6.mp4.

## Data Availability

The data that support the findings of this study are available from the corresponding author upon reasonable request.
