# Supplementary material for: A Microfiber‐Reinforced Janus Hydrogel E‐Skin With Recyclable Feature for Multimodal Sensing and Gender‐Specific Physiological Monitoring
Source: Adv Sci (Weinh). 2025 Dec 22;13(17):e20336. doi: 10.1002/advs.202520336 (PMC13042942; doi:10.1002/advs.202520336)
Supplement: Supplementary file 1 — Supporting File 1: advs73478‐sup‐0001‐SuppMat.docx. [file ADVS-13-e20336-s007.docx]

Supporting Information

A Microfiber-Reinforced Janus Hydrogel E-Skin with Recyclable Feature for Multimodal Sensing and Gender-Specific Physiological Monitoring

*Yarong Ding,^1^ Yufeng Li,^2^ Shaozhe Tan,^1^ Jiachun Sun,^1^ Xu Yang,^2^ Xuesi Zhang,^1^ Zhenhua Lin,^1^ Zhenyu Li,^1^ Yue Hao,^1^ Yannan Liu,^2^* Yingchun Li,^1,3^* and Jingjing Chang ^1,3^**

*^1^ State Key Laboratory of Wide-Bandgap Semiconductor Devices and Integrated Technology, Faculty of Integrated Circuit, Xidian University, Xi’an 710071, P.R. China.*

*^2^ Shaanxi Key Laboratory of Degradable Biomedical Materials, School of Chemical Engineering, Northwest University, Xi’an 710069, P.R. China.*

*^3^ Advanced Interdisciplinary Research Center for Flexible Electronics, Academy of Advanced Interdisciplinary Research, Xidian University, Xi’an 710071, P.R. China.*

*Corresponding authors: jjingchang@xidian.edu.cn; ycli@xidian.edu.cn; liuyannan@nwu.edu.cn


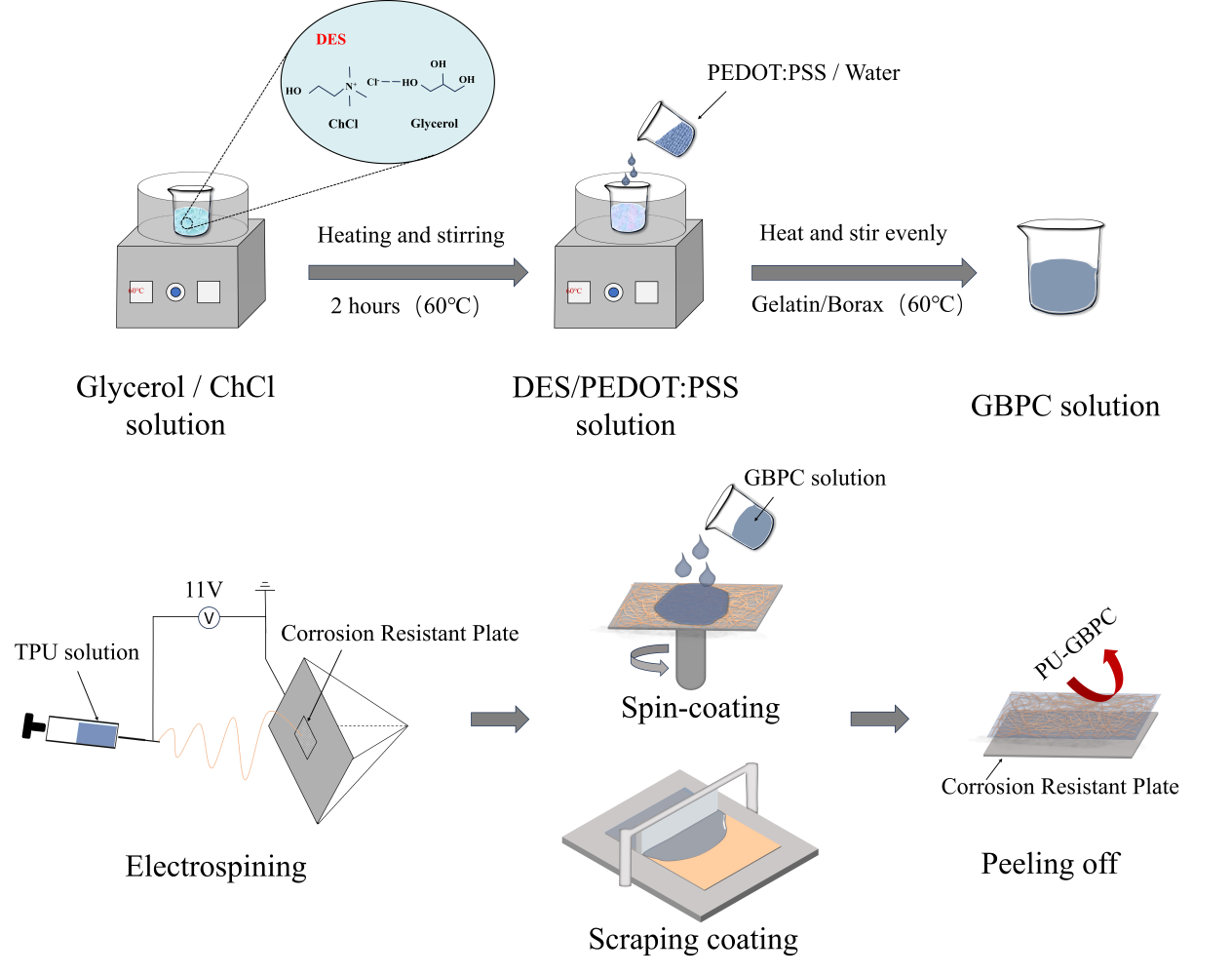
**Figure S1.** Schematic diagram of the preparation process of PU-GBPC hydrogel.


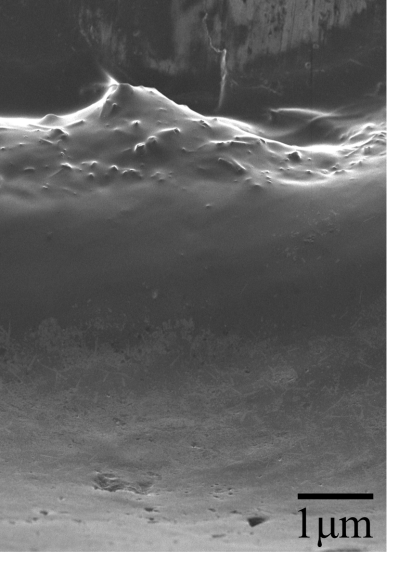


**Figure S2.** SEM cross-sectional image of PU-GBPC composite hydrogel without freeze-drying.


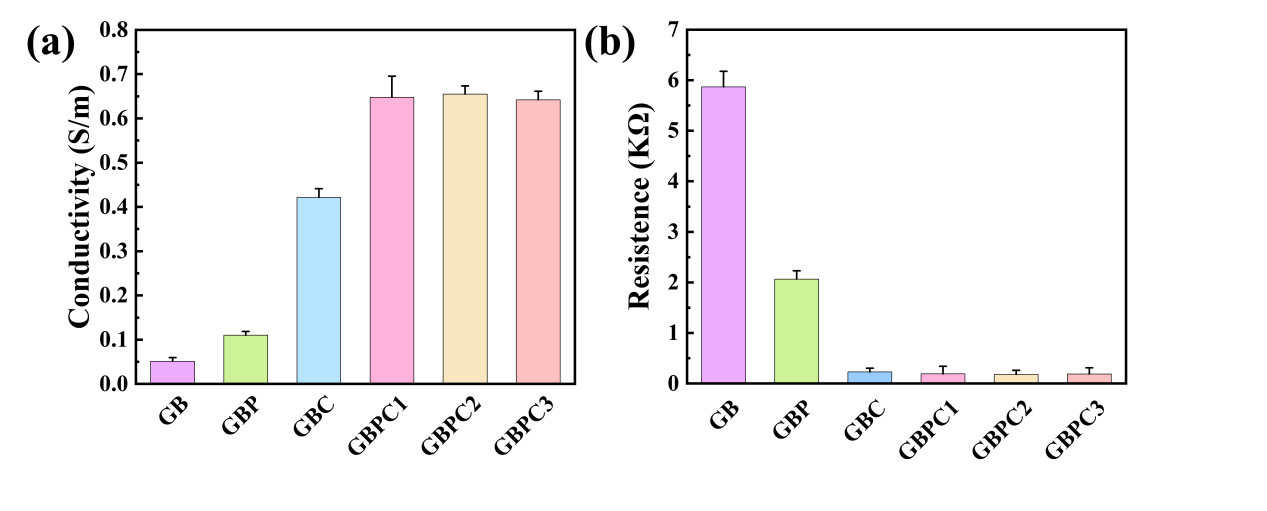


**Figure S3.** The influence of adding conductive substances with different contents on the electrical conductivity of hydrogels.


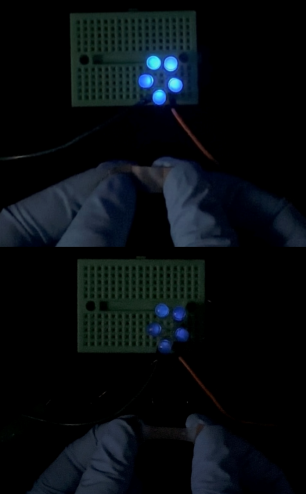


**Figure S4.** The brightness change of the flower-shaped small bulb when connecting the hydrogel in the initial state and the stretched state.


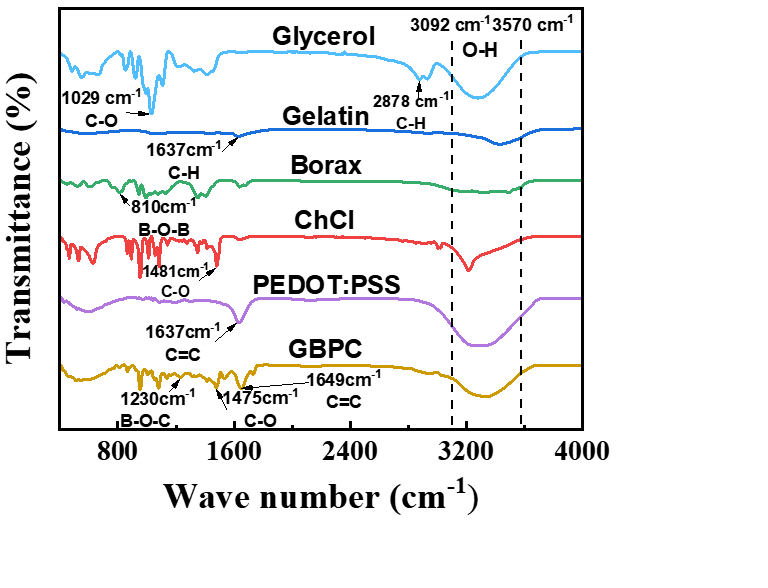


**Figure S5.** Fourier transform infrared (FTIR) spectra of each component in the GBPC hydrogel.

Interactions and structural formation of each component within the GBPC hydrogel system. Fourier transform infrared spectroscopy (FTIR) was used to analyze the interactions and structural formation of the GBPC hydrogel system. The peak detected for glycerol at 1029 cm^-1^ is attributed to C-O stretching, and the signal at 2878 cm^-1^ corresponds to C-H symmetric stretching. The C-O peak in choline chloride (ChCl) shifts from 1481 cm^-1^ to 1475 cm^-1^. In addition, absorption peaks of varying intensities appear in the range of 3092 cm^-1^-3570 cm^-1^, which belong to the symmetric stretching vibration of O-H bonds, and an obvious blue shift of this absorption peak can be observed. These phenomena confirm the formation of deep eutectic solvent (DES). The peak of gelatin at 1637 cm^-1^ corresponding to C-N stretching undergoes a significant blue shift, which can be attributed to the NH_2_ side groups of gelatin and the OH side groups on PEDOT: PSS. Moreover, the C=C stretching of PEDOT: PSS at 1637 cm^-1^ shifts to 1649 cm^-1^, further confirming the embedding of PEDOT: PSS in the gelatin-based hydrogel. The peak at 1230 cm^-1^ in the GBPC hydrogel is attributed to the asymmetric stretching vibration of B-O-C, confirming the formation of borate ester bonds between gelatin and borax. Furthermore, the peak of borax in FTIR at 810 cm^-1^ is caused by the stretching vibration of B(OH)_4_^-^ in B-O and the bending vibration of B-O-B in borax, indicating that there are still small amounts of free B(OH)_4_^-^ and unreacted borax with gelatin. The blue shift of the characteristic broad absorption band in the range of 3092 cm^-1^-3570 cm^-1^ corresponds to the symmetric stretching of O-H, which indicates the existence of strong hydrogen bonds between various components. The synergistic interactions of dynamic borate ester bonds and intermolecular as well as intramolecular hydrogen bonds coordinate to form a robust three-dimensional network, ultimately resulting in the cross-linked structural characteristics of the GBPC hydrogel.


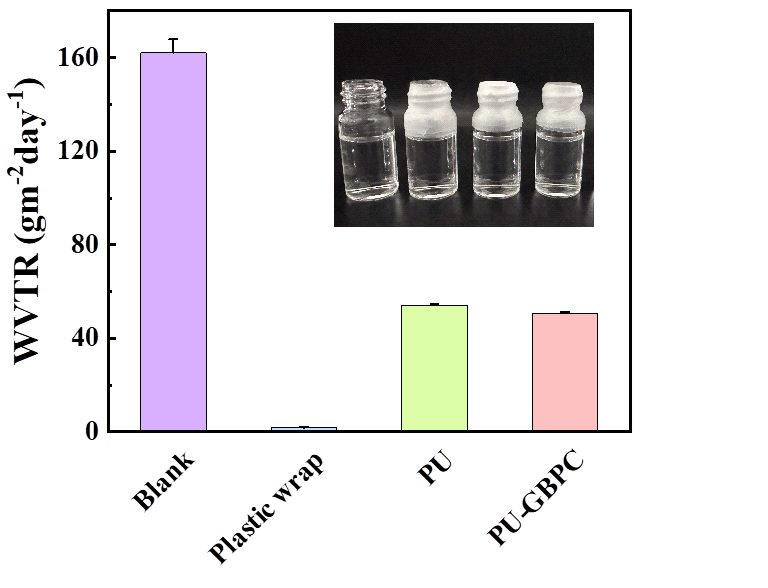


**Figure S6.** Water vapor transmission rate of the PU-GBPC hydrogel sensor and the control group within 12 hours.


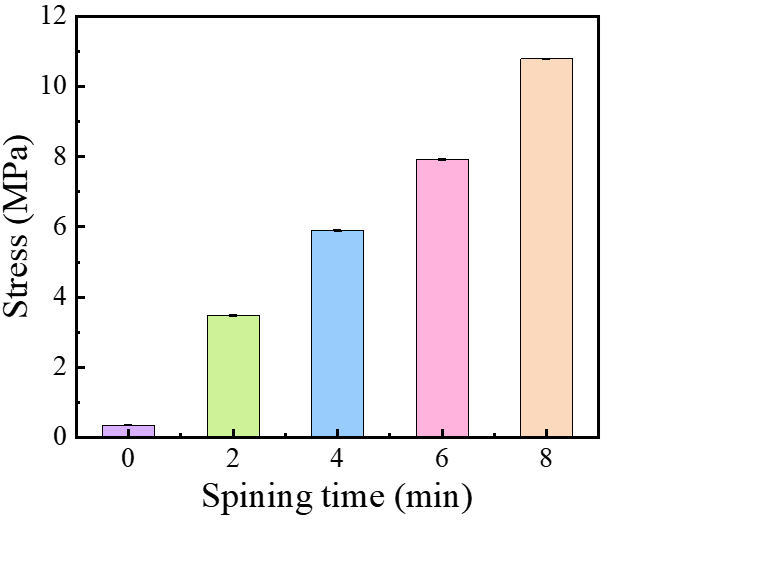


**Figure S7.** Stress diagram of PU-GBPC composite hydrogels with different spinning durations.


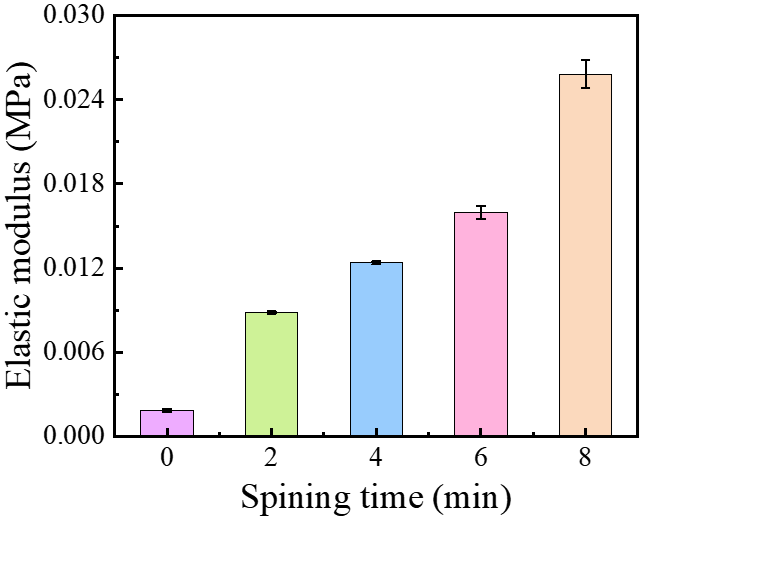


**Figure S8.** Elastic modulus of PU-GBPC composite hydrogels with different spinning durations.


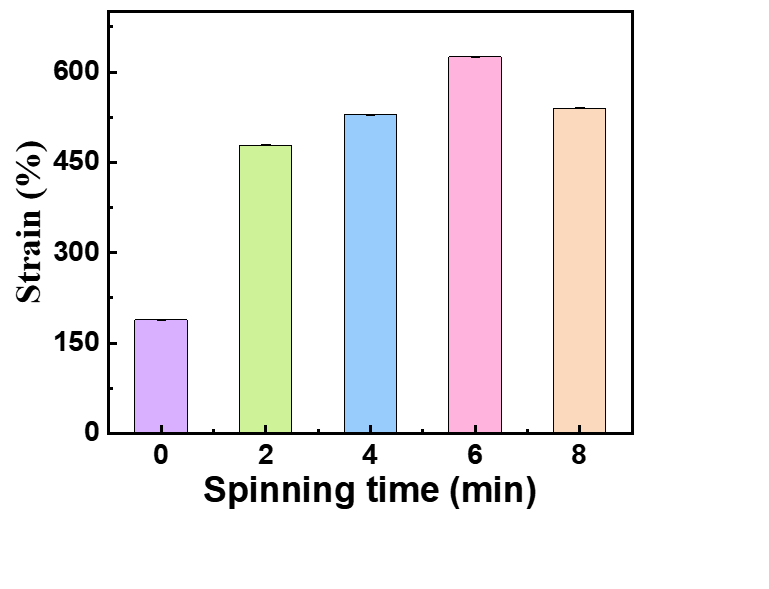


**Figure S9.** Comparison of tensile strength under different spinning times.

**Figure S10.** Stress diagram of PU-GBPC composite hydrogels with different fiber weight fractions.

**Figure S11.** Elastic modulus of PU-GBPC composite hydrogels with different fiber weight fractions.


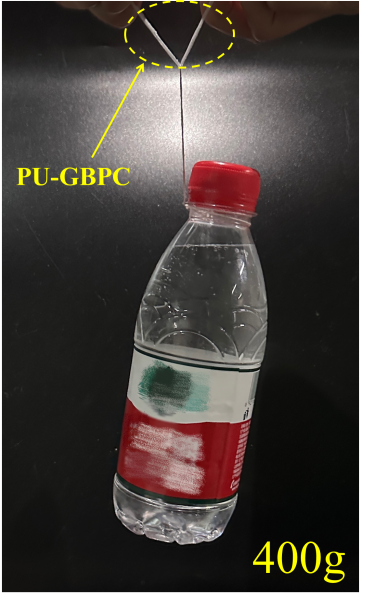


**Figure S12.** The picture shows the mechanical robustness of the PU-GBPC hydrogel.

**Figure S13.** Comparison of tensile strength under different fiber weight fractions.


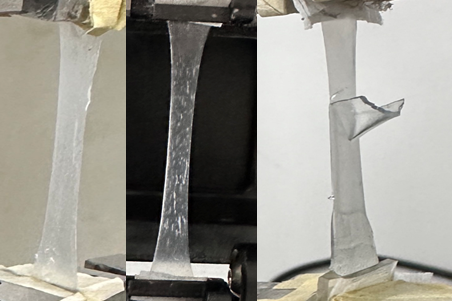


**Figure S14.** Tensile conditions of PU-GBPC with different gel-fiber ratios.


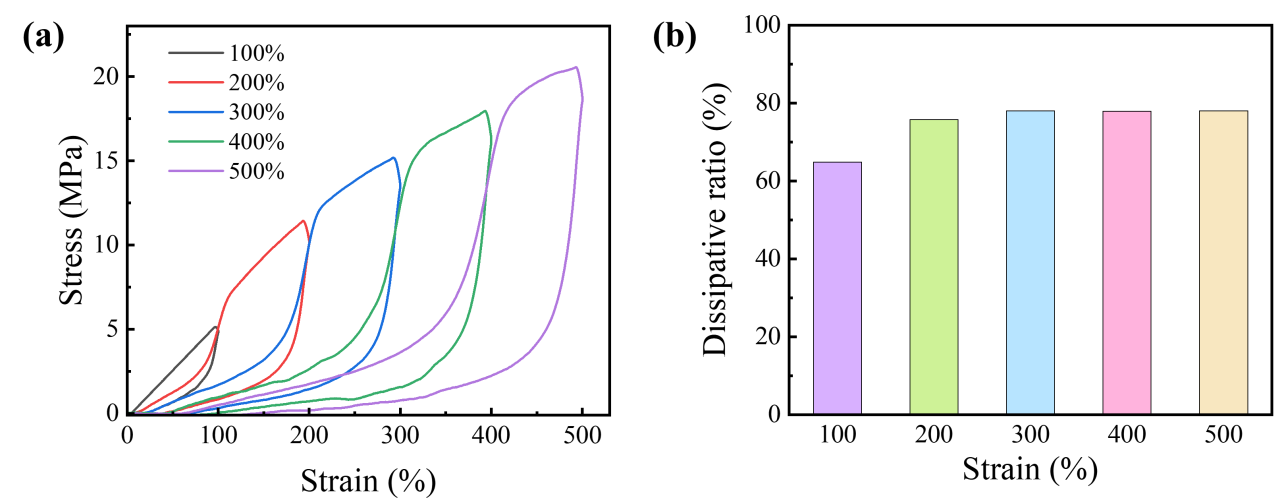


**Figure S15.** Loading-unloading tests of PU-GBPC hydrogels. a) Cyclic tensile curves at 100-500% strain. b) Corresponding dissipation ratios.


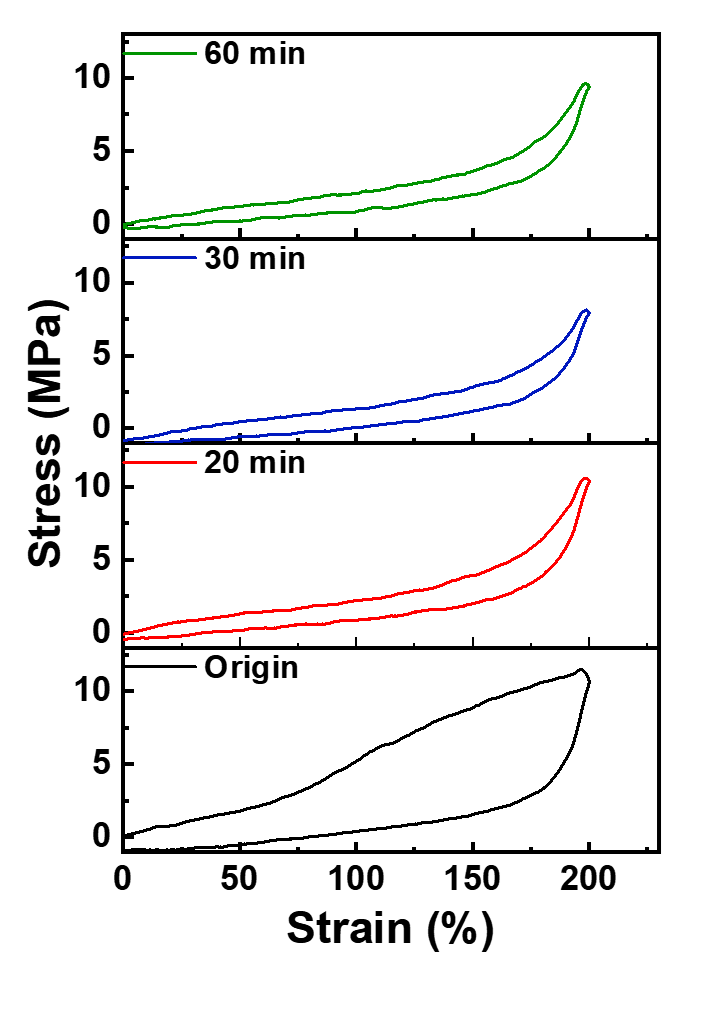


**Figure S16.** Cyclic tensile stress-strain curves of the resting sample.


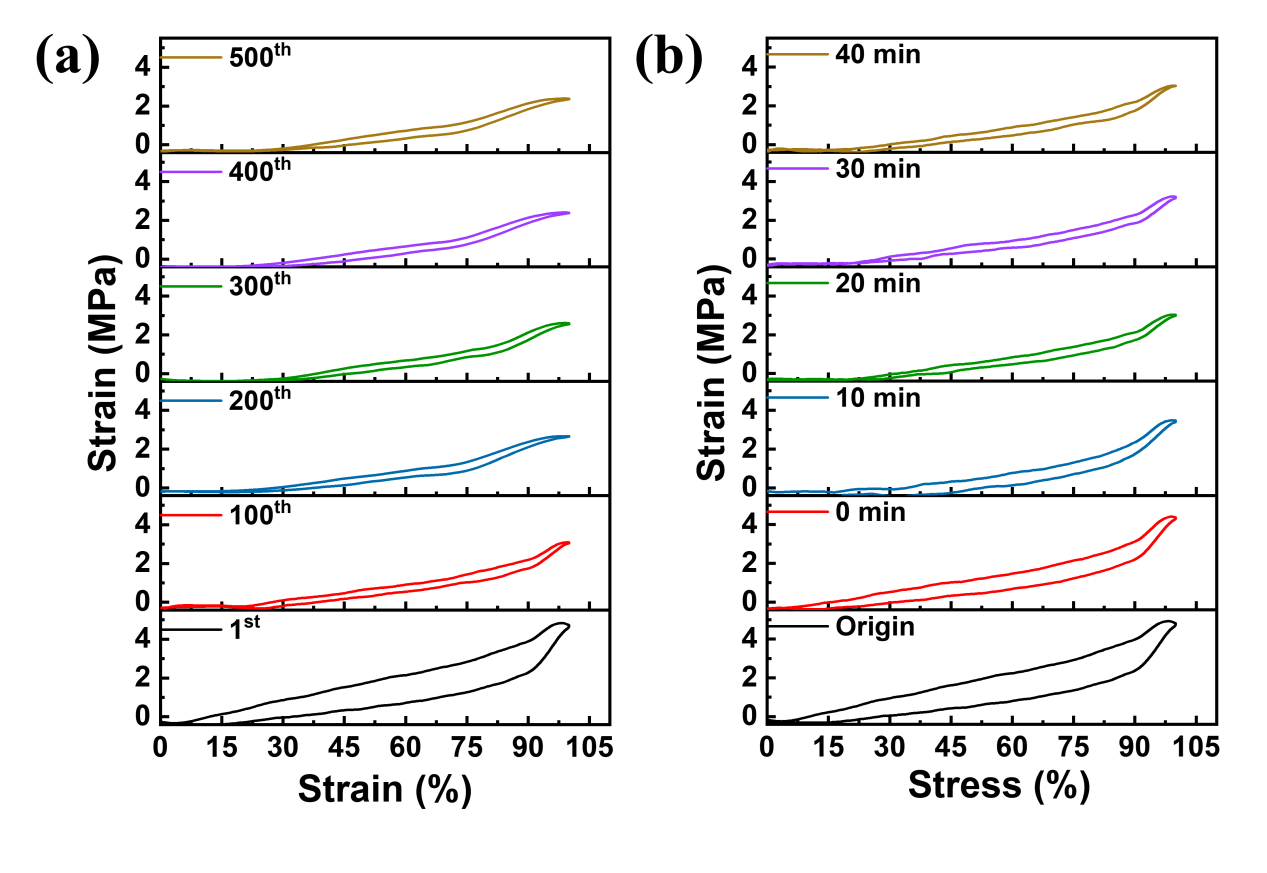


**Figure S17.** Tensile stress-strain curves under different cycles at 100% strain.


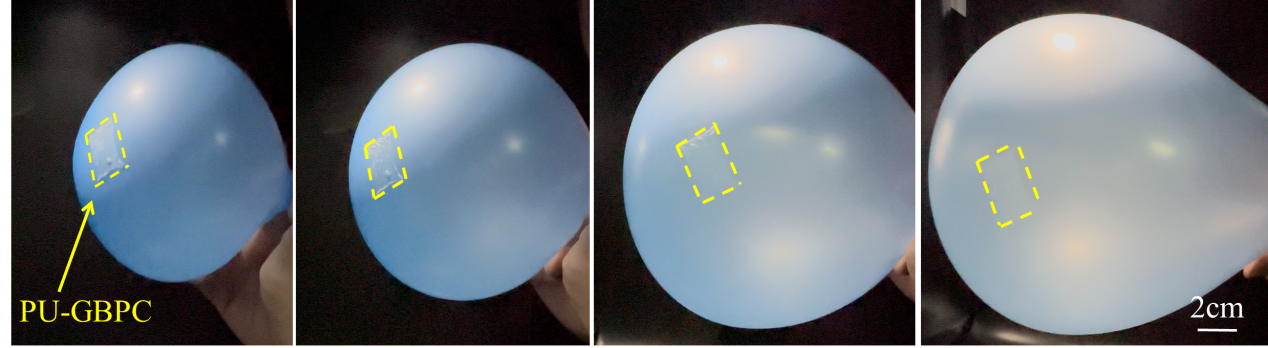


**Figure S18.** The PU-GBPC hydrogel changes with the cyclic stretching of the balloon.


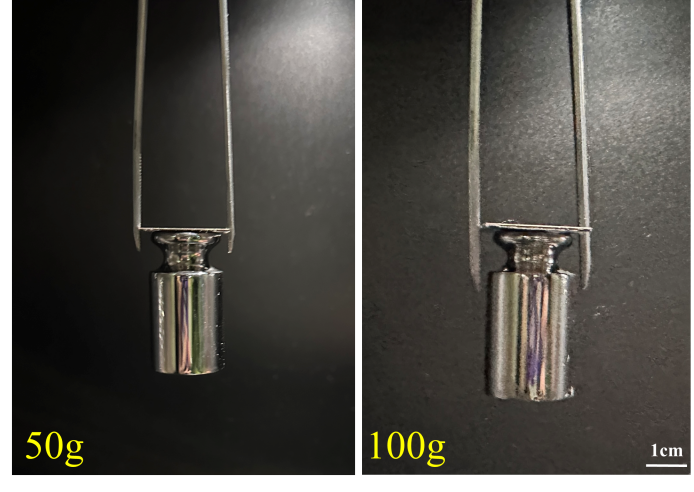


**Figure S19.** Strong adhesion ability of the PU-GBPC gel surface.


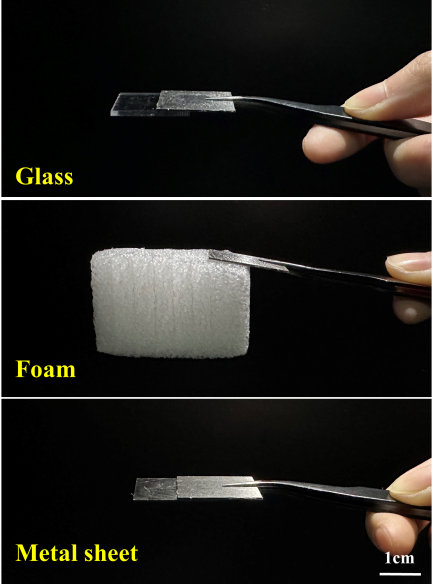


**Figure S20.** The general adhesiveness of the PU-GBPC gel surface, such as to glass, form, and metal sheets.


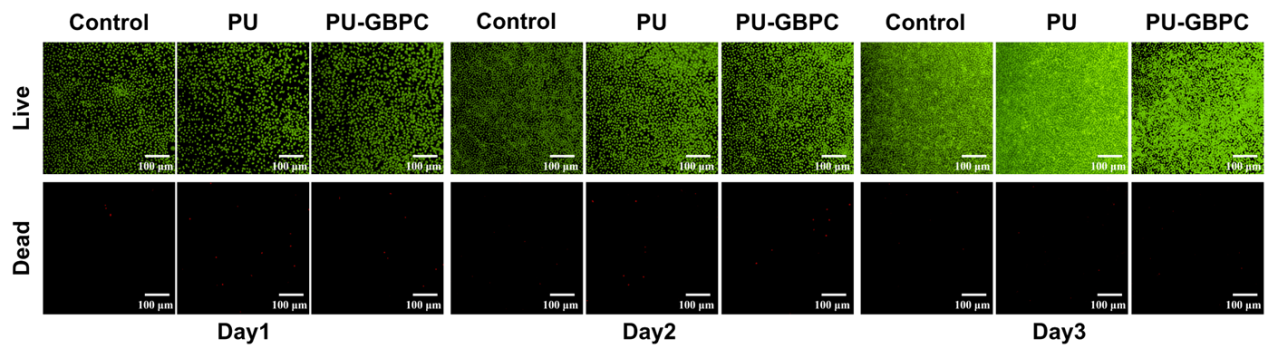


**Figure S21.** Live/dead staing of PU-GBPC hydrogels co-cultured with L929 cells for 24 h, 48 h, 72 h.


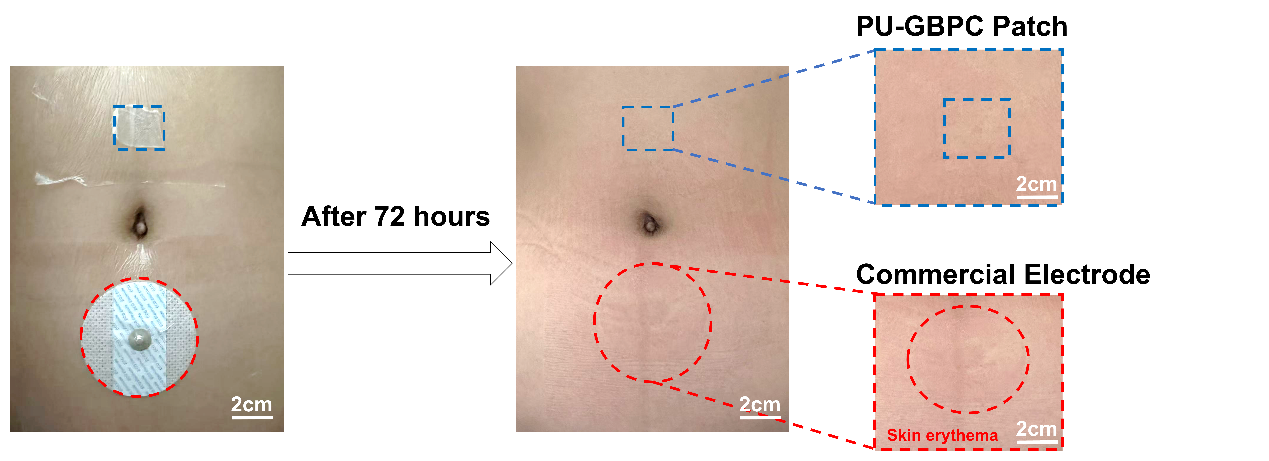


**Figure S22.** Skin conditions after 72 hours of attachment of the PU-GBPC hydrogel sensor and commercial electrode sheets to the abdomen.

**Figure S23.** Comparison of Toughness of the PU-GBPC Hydrogel Sensor Before and After Recycling.

**Figure S24.** Mechanical and electrical stability of the PU-GBPC hydrogel sensor after 10 humidity cycles at 30% and 70%.


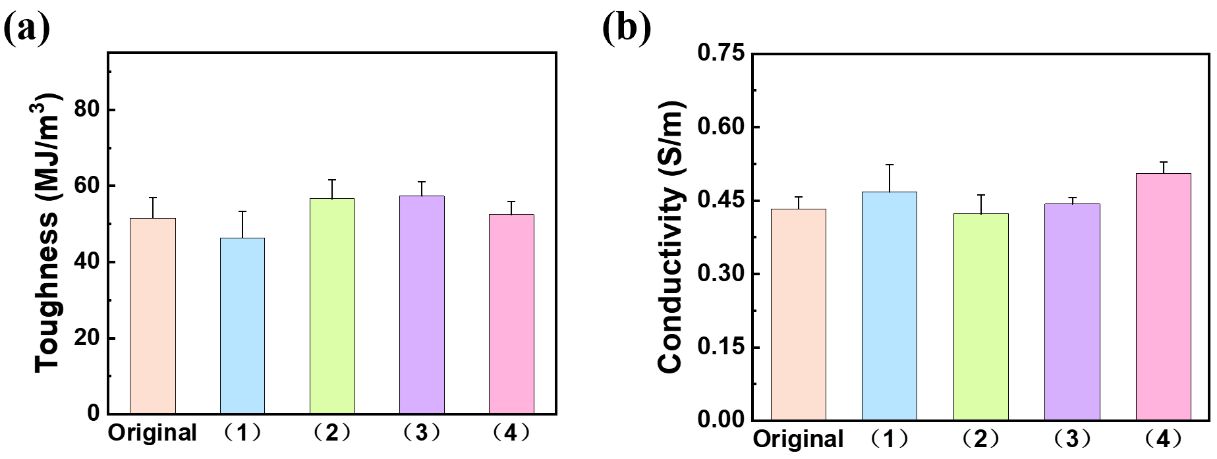


**Figure S25.** The core performances of the hydrogel sensor under four different conditions: (1) exposed on the human skin surface with sweat for 48 hours; (2) 500 friction cycles completed under 30% strain; (3) 100 attachment-separation cycles with human skin; (4) 10 cycles at 30% and 70% humidity: a) Conductivity; b) Toughness.

**Figure S26.** The ∆*R*/*R*_0_ from 25°C to 40°C with a temperature gradient of 5°C and a tensile strain of 200%.


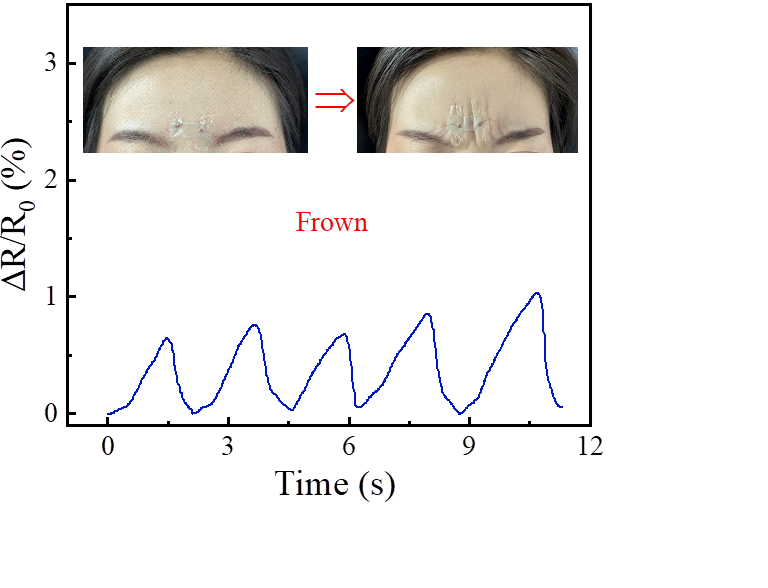


**Figure S27.** The PU-GBPC hydrogel sensor accurately detects the strain of facial muscles when frowning.


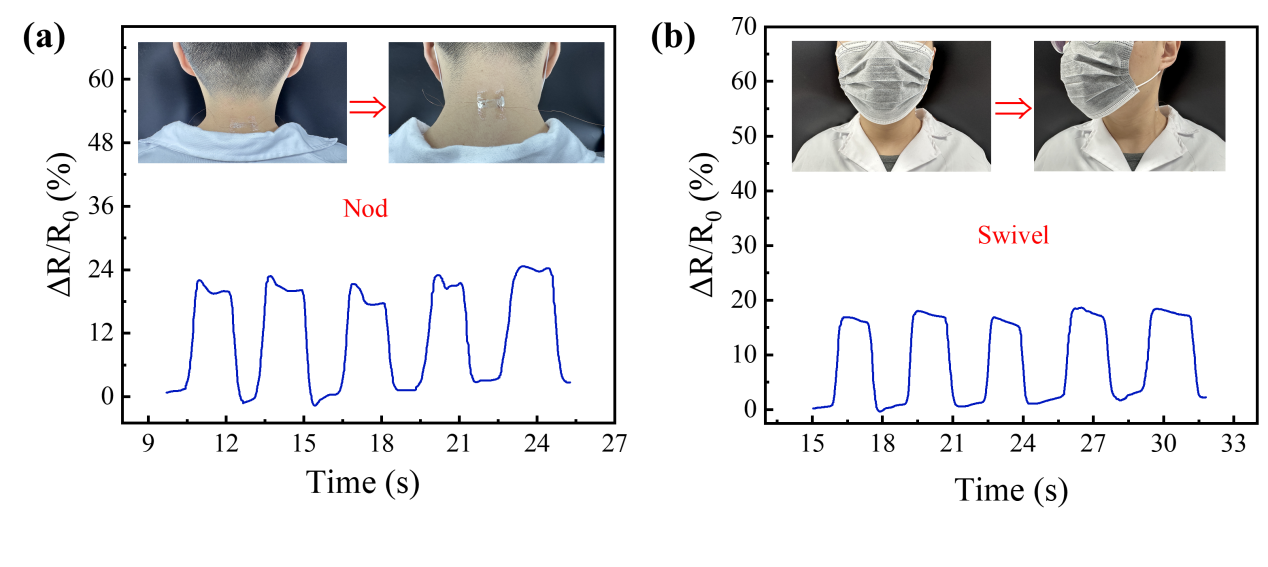


**Figure S28.** Accurate detection of different human body signals by the PU-GBPC hydrogel sensor. a) Nodding. b) Swivel.


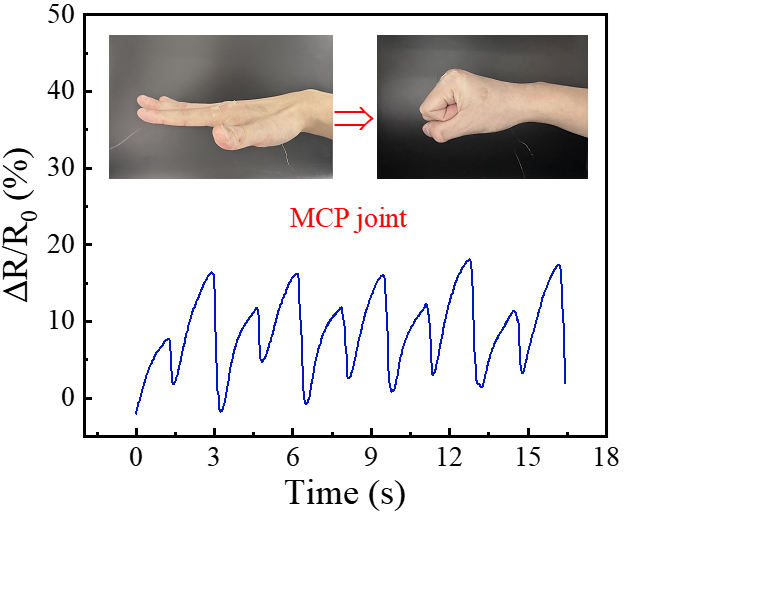


**Figure S29.** Strain detection of the metacarpophalangeal joint by the PU-GBPC hydrogel sensor.


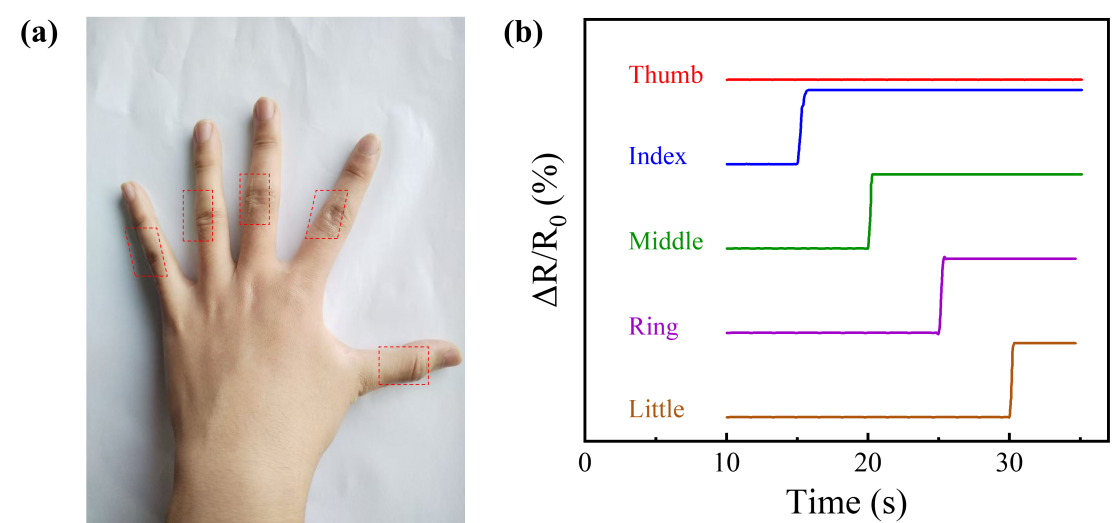


**Figure S30.** The application of the PU-GBPC hydrogel sensor in sign language. a) The PU-GBPC hydrogel sensor is adhered to the finger joints. b) Strain signals of the sign language gesture "in sequence".


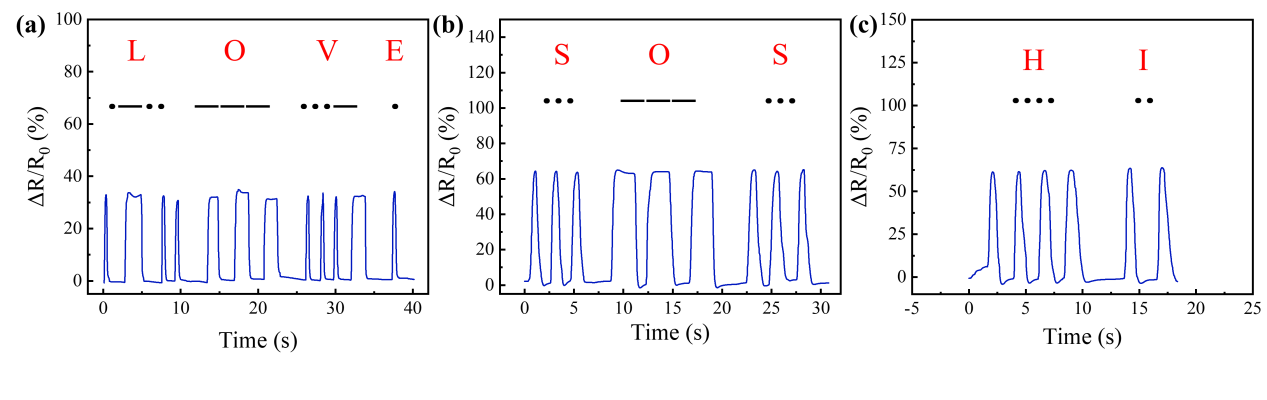


**Figure S31.** PU-GBPC hydrogel sensor for information encryption. a) "LOVE". b) "SOS". c) "HI".


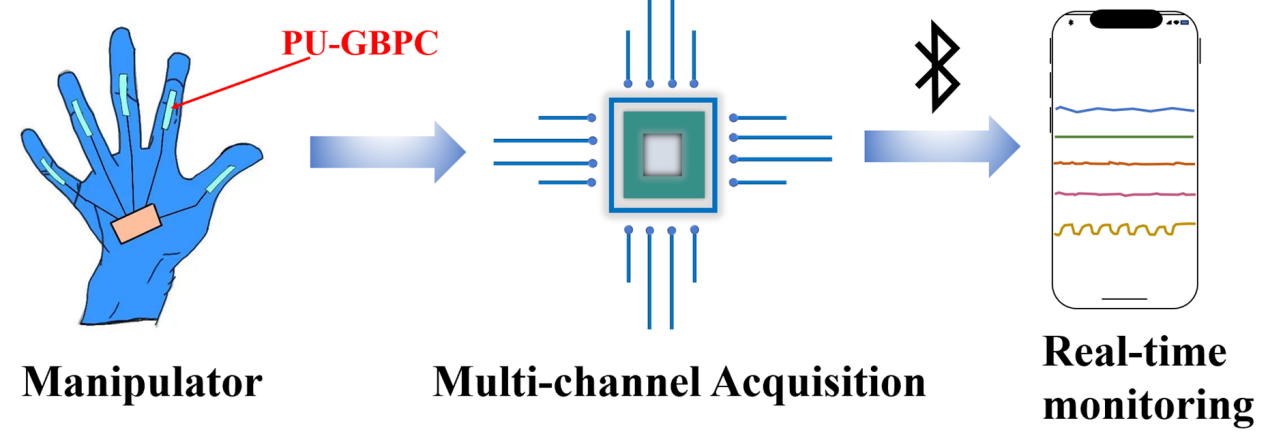


**Figure S32.** System diagram of PU-GBPC hydrogel sensor for mechanical gesture recognition and object grasping.


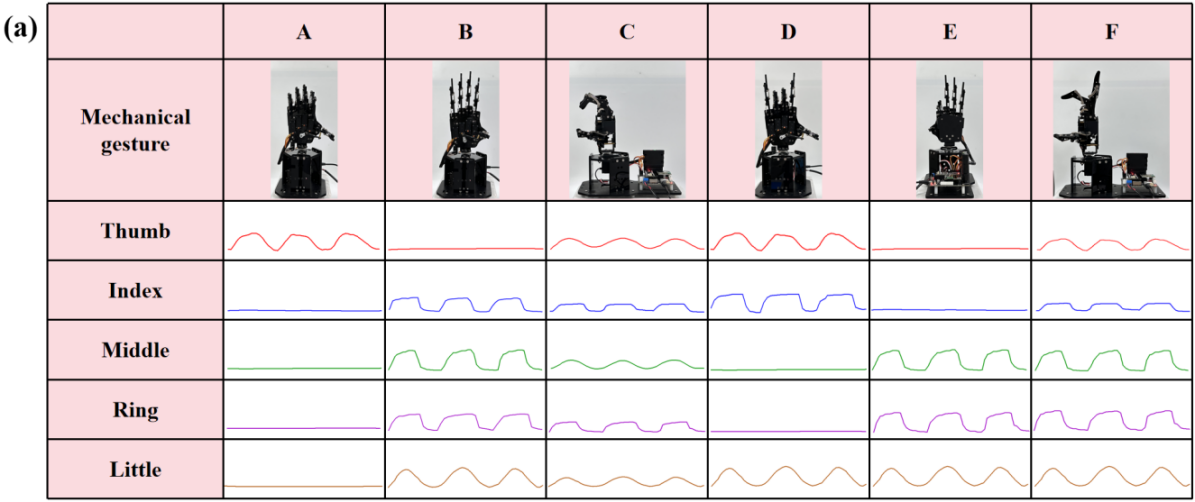


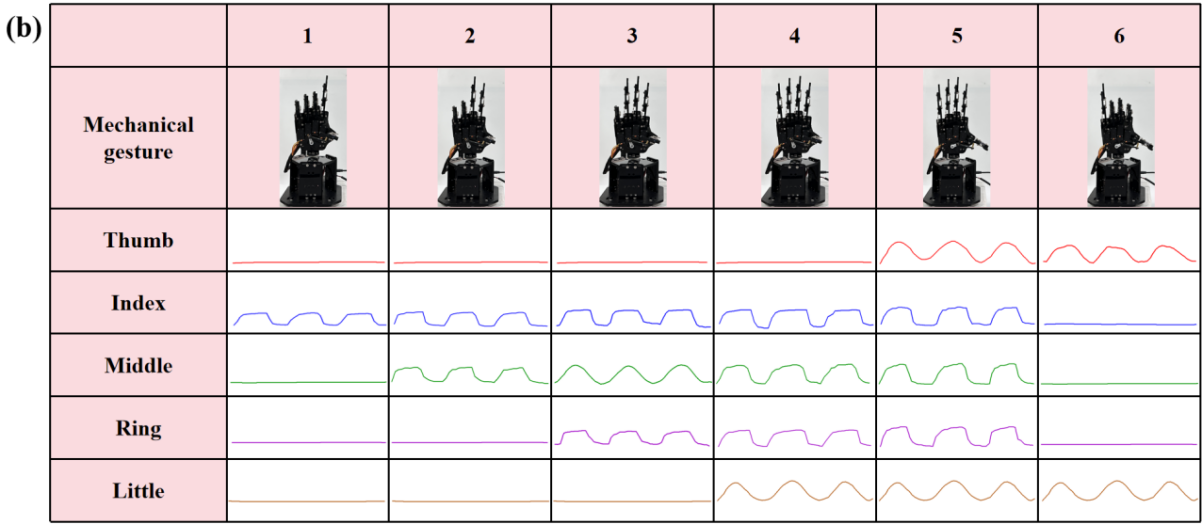


**Figure S33.** A robotic hand connected to a PU-GBPC hydrogel sensor is used to detect the strain signals of five fingers during hand gestures. a) Demonstration of letters A-F. b) Demonstration of numbers 1-6.

**Figure S34.** Curve of the PU-GBPC hydrogel sensor during 6-hour continuous monitoring on a penile mold.


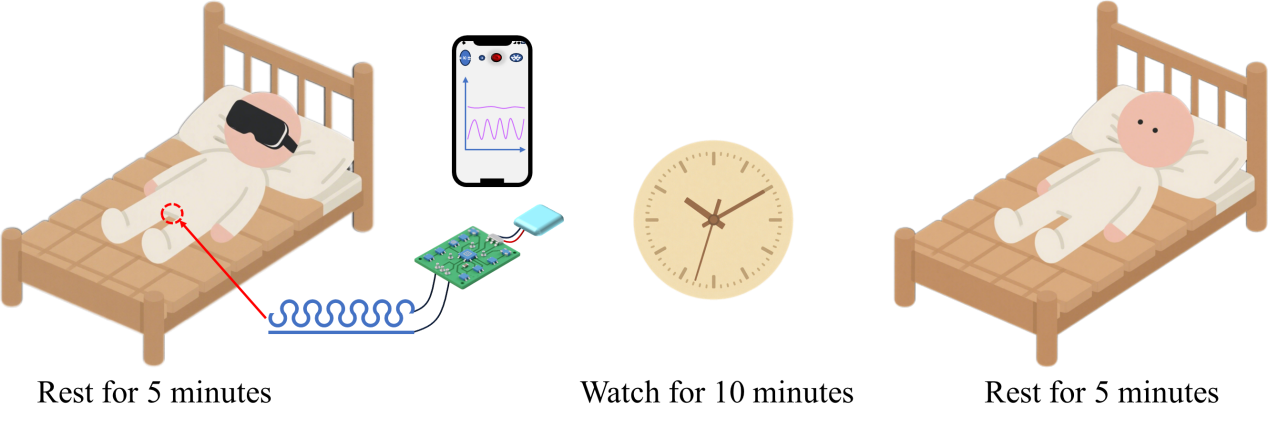


**Figure S35.** Schematic diagram of the penis erection state detection process.

**Table S1.** Hydrogels recipes with varying components.

|  | **PU-spinning time (min)** | **Gelatin(g)** | **Borax(g)** | **PEDOT:PSS(mL)** | **ChCl(g)** | **Glycerol(g)** | **Water(mL)** |
| --- | --- | --- | --- | --- | --- | --- | --- |
| **GB** | / | 1.15 | 0.69 | / | / | 3.68 | 1.05 |
| **GBP** | / | 1.15 | 0.69 | 0.1 | / | 3.68 | 1.05 |
| **GBC** | / | 1.15 | 0.69 | / | 3.0 | 3.68 | 1.05 |
| **GBPC1** | / | 1.15 | 0.69 | 0.07 | 3.0 | 3.68 | 1.05 |
| **GBPC2** | / | 1.15 | 0.69 | 0.1 | 3.0 | 3.68 | 1.05 |
| **GBPC3** | / | 1.15 | 0.69 | 0.1 | 2.8 | 3.68 | 1.05 |
| **PU-GBP** | 6 | 1.15 | 0.69 | 0.1 | / | 3.68 | 1.05 |
| **PU-GBPC** | 6 | 1.15 | 0.69 | 0.1 | 3.0 | 3.68 | 1.05 |

**Table S2.** Comparison table of hydrogel sensor functions. Herein, "NM" stands for "not mentioned", "NO" stands for "no such function", and "YES" stands for "has such function".

| **Ref.** | **Feedstocks** | **Thickness** | **Water loss** | **Adhesion** | **Tensile strength** | **Toughness** | **GF** | **Resolution（%）** | **TCR (% °C ^-1^)** | **Resolution** | **Response time** | **Frost resistance** | **Anti-stretch range** | | **Recyclability** |
| --- | --- | --- | --- | --- | --- | --- | --- | --- | --- | --- | --- | --- | --- | --- | --- |
| [14] | PU nanomesh/PVA/Glycerol/NaCl | <5 μm | - | - | 6 MPa |  |  |  |  |  |  | -20°C |  | | - |
| [15] | PU nanomesh/Gelatin/Borax/Na_2_SO_4_ | ~10 μm | 1% (21 days) | Double-sided | 2.5 MPa | - | - | - | - | - | - | - | 0-696% | | - |
| [18] | DES/SBMA/DA/AA/CTAB | NW | - | Double-sided | 0.25 MPa | 1.58 MJ m^-3^ | - | - | - | - | - | -100°C | 1380-2064% | | - |
| [20] | Gelatin/ChCl Glycerol/Xylitol/D-sorbitol | - | - | Double-sided | 10.25 MPa | 17.34 MJ m^-3^ | 1.59(0-350%)  2.45(>350%) | - | - | - | 318 ms (strain) | -70°C | 0-892.51% | | YES |
| [21] | PVA/DES/DA/AA/Zn | - | - | Double-sided | 25.6 kPa | 101 kJ/m^3^ | 7.22 (0-100%)  11.73 (100-300%)  19.23 (300-500%) | 0.1% | - | - | - | -40°C | 0-1040% | | - |
| [22] | P(EA-co-AA)/(EMI TFSI) | - | - | Double-sided | - | 46.8 kJ/m^3^ | 1.21 (50-500%) |  | -20 (0-20.3°C)  -4.6 (20-40°C)  -0.55 (40-70°C) | 1°C | 220 ms  (strain) | -20°C | 0-10800% | | - |
| [23] | [Bi(NO_3_)_3_·5H_2_O]/MH/AA/PDMS/Glycerol | - | - | Double-sided | 0.37 MPa | 12.14 MJ m^-3^ | 3.76 (0-500%)  15.81 (500-1600%)  57.74 (1600-3100%)  197.68 (3100-5300%) | - | - | - | 137 ms  (strain) | -32.27°C | 0-5417% | | - |
| [37] | PU nanomesh/PVA/AMPS/AAM | ~200 μm | 14% (10 days) | NO | 3.85 MPa | 20.14 MJ m^-3^ | 1.58 (0-100%)  6.68 (nearly 800%) | 0.1% | - | - | - | -80°C | | 0-920% | - |
| [40] | Silica nanofibers/SA/AM/NaCl | - | - | - | 0.3 MPa | 2.98 MJ m^-3^ | 0.82 (0-300%)  2.67 (300-1100%) | - | - | - | - |  | 0-1400% | | - |
| [41] | PAAm/Gelatin/Ammonium | - | <20% (15 days) | - | 899.3 kPa | 2490 kJ/m^3^ | 1.65 (100%)  2.27 (100-250%)  3.06 (250- 400%) | - | - | - | 250 ms (strain) | -20°C | 0-400% | | - |
| **This work** | PU nanomesh/Gelatin/Borax/PEDOT:PSS/DES | ~16.9 μm | 8% (30 days) | Asymmetric | 24.87 MPa | 55.62 MJ m^-3^ | 2.52 (0-650%) | 0.1 | -3.5 (10-25°C)  -1.24 (25-45°C) | 0.1°C | 106 ms (strain) | -80°C | 0-375% | | YES |

PVA: poly(vinylalcohol); AMPS: 2-Acrylamido-2-methylpropanesulfonic acid; AAM: acrylamide; AM: Acrylamide monomer; DES: Deep Eutectic Solvents; SBMA: [2-(methacryloyloxy) ethyl] dimethyl- (3-sulfopropyl) ammonium hydroxide; DA: Dodecyl acrylate; AA: Acrylic acid; CTAB: Hexadecyl trimethyl ammonium bromide; PAAM: Polyacrylamide; MH: [3-(methacryloylamino)propyl]dimethyl(3-sulfopropyl)ammonium hydroxide inner salt; PDMS: Polydimethylsiloxane; SA: Salicylic acid; PEDOT:PSS:poly(3,4-ethylenedioxythiophene):poly(styrene sulfonate).

**Table S3.** Basic Information of Volunteers Provided in Pregnancy and ED Monitoring.

|  | **Gender** | **Height(cm)** | **Age(years)** | **Weight(kg)** | **Pregnancy status** | **Erectile status** |
| --- | --- | --- | --- | --- | --- | --- |
| **1** | Female | 158 | 27 | 47 | Non-pregnant | / |
| **2** | Female | 165 | 23 | 48 | Non-pregnant | / |
| **3** | Female | 168 | 25 | 51 | Non-pregnant | / |
| **4** | Female | 162 | 30 | 67 | Early pregnancy | / |
| **5** | Female | 156 | 29 | 57 | Early pregnancy | / |
| **6** | Female | 160 | 29 | 61 | Early pregnancy | / |
| **7** | Female | 165 | 31 | 68 | Late pregnancy | / |
| **8** | Female | 160 | 29 | 83 | Late pregnancy | / |
| **9** | Female | 159 | 27 | 76 | Late pregnancy | / |
| **10** | Male | 185 | 25 | 73 | / | Normal |
| **11** | Male | 183 | 24 | 65 | / | Normal |
| **12** | Male | 180 | 25 | 79 | / | Normal |
| **13** | Male | 175 | 31 | 82 | / | Psychogenic ED |
| **14** | Male | 172 | 39 | 75 | / | Psychogenic ED |
| **15** | Male | 182 | 33 | 77 | / | Psychogenic ED |
| **16** | Male | 177 | 38 | 6 | / | Organic ED |
| **17** | Male | 180 | 35 | 71 | / | Organic ED |
| **18** | Male | 180 | 39 | 76 | / | Organic ED |

**Video S1:** Conductivity and stretchability of the PU-GBPC hydrogel sensor.

**Video S2:** Janus characteristics of the PU-GBPC hydrogel sensor.

**Video S3:** Strain consistency of PU-GBPC hydrogel before and after recovery.

**Video S4:** Stretchability of the PU-GBPC hydrogel sensor at room temperature and -80°C.

**Video S5:** (Spherical mold) Finite element analysis comparing the stress distribution of annular and serpentine structures under 15% strain condition.

**Video S6:** (Columnar mold) Finite element analysis comparing the stress distribution of annular and serpentine structures under 15% strain condition.
